# Deciphering the Mysterious Relationship between the Cross-Pathogenetic Mechanisms of Neurodegenerative and Oncological Diseases

**DOI:** 10.3390/ijms241914766

**Published:** 2023-09-29

**Authors:** Yulia Aleksandrova, Margarita Neganova

**Affiliations:** 1 Institute of Physiologically Active Compounds at Federal Research Center of Problems of Chemical Physics and Medicinal Chemistry, Russian Academy of Sciences, 142432 Chernogolovka, Russia; aleksandrova@ipac.ac.ru; 2 Arbuzov Institute of Organic and Physical Chemistry, FRC Kazan Scientific Center, Russian Academy of Sciences, 420088 Kazan, Russia

**Keywords:** neurodegenerative diseases, Alzheimer’s disease, cancer, molecular mechanisms of pathogenesis, oxidative stress, epigenetics, metabolism, drugs

## Abstract

The relationship between oncological pathologies and neurodegenerative disorders is extremely complex and is a topic of concern among a growing number of researchers around the world. In recent years, convincing scientific evidence has accumulated that indicates the contribution of a number of etiological factors and pathophysiological processes to the pathogenesis of these two fundamentally different diseases, thus demonstrating an intriguing relationship between oncology and neurodegeneration. In this review, we establish the general links between three intersecting aspects of oncological pathologies and neurodegenerative disorders, i.e., oxidative stress, epigenetic dysregulation, and metabolic dysfunction, examining each process in detail to establish an unusual epidemiological relationship. We also focus on reviewing the current trends in the research and the clinical application of the most promising chemical structures and therapeutic platforms that have a modulating effect on the above processes. Thus, our comprehensive analysis of the set of molecular determinants that have obvious cross-functional pathways in the pathogenesis of oncological and neurodegenerative diseases can help in the creation of advanced diagnostic tools and in the development of innovative pharmacological strategies.

## 1. Introduction

Studies conducted over the past three decades show that the treatment of oncological diseases and neurodegenerative disorders remains very limited [1,2]. This is primarily due to the numerous adverse effects of the existing drugs as a result of their systemic action due to the lack of knowledge about the etiopathogenesis of these disorders. As a result, in recent years, there has been a fundamental revision of the currently prevailing traditional therapeutic paradigms with the aim of developing the most promising pharmacological approaches.

Despite the fact that malignant neoplasms and neurodegenerative disorders are considered to be two completely different groups of diseases, and their epidemiological association is extremely complex, in both cases, the mechanisms of cellular regulation are disturbed. Moreover, if the occurrence and progression of cancers are associated with uncontrolled cell proliferation [3], then dementia, on the contrary, is characterized by brain atrophy as a result of extensive neuronal death [4].

The growing interest in the problem of determining the relationship between oncological and neurodegenerative diseases in order to find effective therapeutic agents has allowed us to identify a number of common pathological cascades. These are associated with the dysregulation of signaling pathways and changes in the expressions of genes and proteins [5]. Such changes mainly occur in antagonistic directions. For example, long before the first symptoms of Alzheimer’s disease appear in non-neuronal brain cells and on the periphery, there is an overexpression and aberrant activity of the incorrectly folded protein p53 [6], while its functional insufficiency is associated with tumor transformation and the progression of a wide range of malignant neoplasms [7]. Meanwhile, the reduced level of the apoptosis regulator Bcl-2 observed in neurodegenerative diseases [8,9] reflects the vulnerability of neuronal cells to death [10]. This phenomenon is the opposite to the overexpression of the anti-apoptotic protein in oncological diseases [11,12], leading to the increased survival of tumor cells and the development of their chemo- and radioresistance [13,14,15].

However, special attention should be paid to the processes involved in the etiopathogenesis of the diseases, which have significant pathophysiological correlations with each other. The list of such processes is dominated by oxidative stress and mitochondrial dysfunction [16,17,18,19,20,21], alterations in the bioenergetic cell metabolism [22,23], and epigenetic dysregulation [24,25]. To confirm this, an inverse correlation between cancers and neurodegeneration may be considered [26] when the prevalence of malignant neoplasms is significantly reduced in people with various forms of neurodegenerative disorders receiving maintenance therapy (approximately 70%) [27]. Meanwhile, among patients with a history of successfully cured cancer, there is a reduction in the risk of developing Alzheimer’s disease (approximately 50%) [27], Parkinson’s disease [28], and other dementia syndromes.

In this regard, having analyzed many experimental and review works conducted by leading researchers worldwide, we attempted to establish complex biological links between the pathogenesis of malignant neoplasms and neurodegenerative disorders. Since the data on the general mechanisms of the development of these diseases suggest the introduction of new therapeutic strategies, we also provided a detailed definition of potential targets for the action of promising therapeutic agents. All this can lead to a better understanding of their etiopathological mechanisms, as well as contribute to the discovery of promising new areas in the development of effective pharmacological strategies.

## 2. Oxidative Stress and Mitochondrial Dysfunction in Oncological and Neurodegenerative Diseases

### 2.1. Concept of Oxidative Stress and Sources of Free Radicals

For almost four decades, in the research community in experimental and clinical medicine, there has been a genuine interest in the role of reactive oxygen species (ROS) in the treatment of various diseases. Among the most important are both radical molecules (hydroxyl radicals (OH^•^), superoxide anion radicals (O_2_^•−^), and peroxyl radicals (ROO^•^, RCOO^•^)), and non-radical molecules (hydrogen peroxide (H_2_O_2_), singlet oxygen (^1^O_2_), nitric oxide (NO), and peroxynitrite (ONOO^−^)) [29].

The initial integration of reactive oxygen species into biomedical concepts was associated exclusively with their toxic effects and irreversible functional changes in various disorders. Since then, knowledge of the cell redox balance has developed rapidly. Nowadays, the dual role of reactive oxygen species in the functioning of live systems is well known. Acting as secondary messengers, reactive oxygen species at the basal level (in low or moderate concentrations) regulate the flow of numerous intracellular signaling cascades [30,31,32,33]. In turn, their overproduction and cumulative production in biological systems as a result of endogenous or exogenous effects are toxic. It makes ROS important mediators of nonspecific damage to cellular structures (known as oxidative stress), including proteins, lipids, carbohydrates, and nucleic acids [34]. In other words, the concept of oxidative stress includes disturbances in the homeostasis of free radicals that occur as a result of the overproduction of reactive oxygen species and a deficiency of antioxidant enzymes that can detoxify reactive intermediates and repair damage [35].

The formation of reactive oxygen species is a consequence of oxidative phosphorylation, and mitochondrial ROS play a crucial role in a number of redox signaling processes and in the formation and progression of various pathological conditions. It is believed that mitochondria are the main contributors to the intracellular production of reactive oxygen species [36].

Since the delicate balance between the levels of free radicals is a key aspect in the normal functioning of the body, the processes related to oxidative stress are considered to be optimal therapeutic targets for the action of potential drug agents.

### 2.2. Free Radical Theory of the Occurrence of a Pathological Condition in a Cell

Currently, there are many theories and hypotheses that attempt to explain the driving forces behind the onset and progression of malignant neoplasms and neurodegenerative disorders. One of the most popular and fundamental postulates is the free radical theory, which interprets these pathological processes at the molecular level.

The free radical theory was first suggested in 1956 by Denham Harman to explain the attack of cellular components by free radicals produced by mitochondria as byproducts during normal metabolism [37]. It is now known that these organelles are one of the key sources of reactive oxygen species formed as a result of electron leakage in the electron transport chain [38].

Owing to ongoing discoveries in this area, 16 years later, Harman made an important statement, arguing that the lifespans of mammals depend on the rate of oxygen utilization and proposing a mitochondrial-free radical theory of aging [37]. Thus, the main postulate of Harman’s theory states that “the prolonged presence of these unstable and reactive molecules in the system can lead to a direct or indirect damage to cellular components and connective tissues” [37]. Since then, Harman’s theory has led to the idea that the removal of such molecules will reduce cell damage and, as a result, can slow down the pathological process. Much effort has been made to verify the validity of this position, and, to date, it has been confirmed in a large number of works demonstrating the abilities of antioxidant therapy, leading to an increase in the lifespan of model organisms [39].

It is well known that the course of various types of neurodegenerative disorders is accompanied by progressive modifications or the deterioration of brain metabolism. One of the main causes of this phenomenon, oxidative stress, affects a number of metabolic pathways [40], and evidence of the involvement of free radicals in cognitive impairment has been obtained from patients suffering from neurodegeneration. In Alzheimer’s disease, redox-mediated damage to various biomolecules has often been reported [41,42,43,44,45,46,47,48,49,50,51,52]. Thus, in the search for the mechanisms underlying this disorder, in addition to the dominant hypothesis of the amyloid cascade, an alternative explanation for the pathogenesis of this disease was proposed, which consisted of the relationship between mitochondrial dysfunction and the hyperproduction of reactive oxygen species. These signs are commonly exhibited in patients with this neuropathology [41]. High levels of biomarkers of oxidative stress in blood plasma and urine have been found in both animal models [42,43,44,45,46,47] and patients with Alzheimer’s disease [48,49,50,51], which correlates with the aggregation and deposits of β-amyloid. In addition, a proteomic study has shown that the higher risk of developing neurodegenerative disorders in patients with Down syndrome can be explained by their higher susceptibility to damage caused by oxidative stress [52].

Oxidative damage to lipids plays a key role in the hyperproduction of reactive oxygen species in the brain [53]. Their high contents in this organ (36–40% in gray matter, 49–66% in white matter, and 78–81% in the myelin sheath) are well known, as is the need for significant oxygen consumption by nerve cells [54]. During this process, lipids are exposed to ROS; as a result, through the mechanism of a chain reaction of free radicals, corresponding products are formed [55]. Interestingly, histological studies show the co-localization of lipid peroxidation products and β-amyloid plaques in the brains of patients with Alzheimer’s disease [56,57]. Thus, the evaluation of the malondialdehyde content showed significantly higher levels of this marker in the hippocampus, occipital and temporal cortices, and serum and plasma [58,59,60] in people with Alzheimer’s disease compared to a healthy group of people. Similar results were obtained in the analysis of levels of 4-hydroxy-2-trans-nonenal (4-HNE), isoprostanes and neurostans, acroleins, oxidized low-density lipoproteins, phospholipids, and hydroperoxides [60,61,62,63].

Because mitochondria are the main sources of reactive oxygen species in the central nervous system, these organelles take a leading position in the pathological hierarchy of neurodegenerative diseases [64]. It has been proven that almost all aspects of mitochondrial function are disrupted in Alzheimer’s disease. The current hypothesis of the mitochondrial cascade, explaining the onset and progression of AD, states that the dysfunction of these organelles affects the expression and processing of the β-amyloid precursor protein (APP), contributing to the oligomerization of pathological forms of the peptide [65,66]. Later, it was discovered that β-amyloid is itself a source of oxidative stress, and the incubation of neurons with oligomers of peptide Aβ_1-42_ leads to lipid peroxidation, as evidenced by high levels of the marker of this process, 4-HNE [67]. Interestingly, 4-HNE levels are proportional to the degree of neuronal damage [68]. In addition, it has been shown that oxidative stress caused by amyloid β (mainly as a result of the formation of metalloamyloid complexes of protein with redox-active metals, i.e., copper, zinc, and iron) leads to the disruption of mitochondrial functioning [69] and contributes to the depolarization of the organelle membrane and the development of the phenomenon of excitotoxicity. At the same time, the alleviation of mitochondrial dysfunction leads to the attenuation of the pathological signs of Alzheimer’s disease. This allows us to consider targeting damaged mitochondria as a key strategy for reducing oxidative stress in models of this disease.

It is obvious that, initially, the free radical theory was associated with aging and degenerative processes occurring in the brains of patients with various types of dementia. With the development of interdisciplinary cooperation between the pharmaceutical and biochemical fields of science with the aim of better understanding the specificity of redox processes, the levels of reactive oxygen species began to attract the attention of researchers in the field of cancer.

Traditionally, the increase in free radical levels has been considered a promising therapeutic strategy for inducing oxidative stress, damaging cellular components, killing tumor cells by inducing apoptosis [70], and triggering autophagy [71]. However, the use of such prooxidant therapies can lead to a number of adverse effects in relation to a healthy microenvironment. For example, the stimulation of oxidative stress via the well-known cytostatic alkylating effect of cisplatin occurs not only in tumor cells but also in cells of normal origin, which is the cause of a common adverse effect of the drug: ototoxicity [72]. Similar to cisplatin, for doxorubicin, the conventional anthracycline antibiotic that leads to the direct generation of hydrogen peroxide and, as a result, the subsequent depolarization of the mitochondrial membrane and caspase-dependent cell death, non-selective localization was found in the mitochondria of untransformed cells; this causes its high cardiotoxicity and imposes serious limitations on its clinical use [73].

Such examples allowed for a revision of the existing concept; therefore, further studies became the basis for the assumption that reactive oxygen species are the driving factor of oncogenesis [74]. Thus, it was found that the elevated levels of ROS shown in various types of tumor cells are, on the contrary, oncogenic due to the damaging effects of DNA, proteins, and lipids, which contribute to genetic instability and the emergence and progression of oncogenesis [32]. This is shown by a significant increase in the above-mentioned markers of oxidative stress in the biological material of patients with malignant neoplasms. In particular, an increase in malondialdehyde is found in carcinoma of the breast [75,76], lung [77], prostate [78,79,80] and bladder [81,82], oral cavity, and oropharynx [83,84], the levels of which correlate with the clinical stages of cancer, reaching their maximum in stages III and IV [85], which indicates the direct role of oxidative stress in the progression of diseases. Damage in DNA malignancies as a result of free radicals is confirmed by an increase in excreted 8-hydroxy-2-deoxyguanosine (8-OHdG), which plays an important role in carcinogenesis for the transformation of healthy cells to tumor cells [86]. Studies show that high levels of 8-OHdG in urine, plasma, and serum are prognostic factors in carcinoma of the esophagus [86,87], ovaries [88,89], and large intestine [90,91,92]. Such ectopic accumulations of reactive oxygen species and the development of permanent oxidative stress lead to dedifferentiation [93] and abnormal cell growth [94,95], metastasis [96], the emergence of resistance to apoptosis (in particular, due to the increased glucose metabolism and adaptation to hypoxic conditions) [97], angiogenesis [98], and the generation of oncogenic mutations [99].

Thus, despite all of the inconsistencies detailed above, to date, both an inhibition of free radical reactions and a stimulation of the production of reactive oxygen species for the specific destruction of transformed cells are considered to be promising strategies for antitumor therapy. However, the use of the latter as a promising therapeutic tool should have a strict selective focus on tumor cells.

### 2.3. Dysfunction of the Cell Antioxidant Defense System

Because the generation of reactive oxygen species is an inevitable phenomenon, not only in pathological conditions but also in normal conditions, living organisms have evolutionarily formed the intrinsic antioxidant defense system, aimed at the strict regulation of redox homeostasis and striving to eliminate excessive amounts of reactive oxygen species without the inhibition of their useful role [100,101].

The cellular system of endogenous antioxidant defense consists of a number of components, the protective mechanisms of which function in two main directions: (1) the elimination of free radicals and reactive forms with the enzymes superoxide dismutase, catalase, glutathione peroxidase, and glutathione reductase [102] and (2) the elimination of free radicals by the electron donor glutathione (GSH) [103].

The first line of the intrinsic antioxidant system, which plays a key role in the protective mechanisms of the cell, consists directly of the above-mentioned enzymes, which act by binding superoxide and other peroxides. The enzymes convert them into stable compounds and thus have an important biological defense role against attacks by ROS [101].

The enzyme superoxide dismutase, which catalyzes the dismutation reaction of the superoxide anion radical into hydrogen peroxide, is localized in the cytosol and mitochondria [104]. Furthermore, the resulting hydrogen peroxide is converted into water and oxygen as a result of the enzymatic activity of catalase [105]. Despite the fact that this antioxidant is abundantly localized in various compartments of cells and is considered the main antioxidant [106], catalase is not found in mitochondria. Another enzyme, glutathione peroxidase, is needed for the degradation of hydrogen peroxide molecules in these organelles, where its main activity is implemented by converting H_2_O_2_ into two H_2_O molecules. It is known that glutathione peroxidase is the first enzyme activated under high ROS levels [107]. In addition to H_2_O_2_, glutathione peroxidase also converts peroxides and hydroxyl radicals to non-toxic compounds by sequentially oxidizing reduced glutathione to glutathione disulfide, which is reduced to GSH under the action of glutathione reductase [108]. One of the most important functions of glutathione reductase is to maintain the ratio of reduced and oxidized forms of glutathione [109].

Glutathione is one of the main non-enzymatic antioxidants that is involved in many cellular functions by acting as a free radical scavenger [110]. It is synthesized in the cytosol and then distributed to almost all cell compartments, including the mitochondrial matrix, where it reacts with ROS and prevents apoptosis [111]. Under physiological conditions, thiol-reduced glutathione (GSH) is the basic form, which is present at much higher concentrations than its disulfide-oxidized form (GSSG); meanwhile, under oxidative stress conditions, the ratio shifts towards GSSG [112].

Various age-related and metabolic diseases are closely associated with abnormal levels of endogenous antioxidants [19,113]. Thus, in the biological materials of patients with cervical carcinoma, significantly lower activity levels of superoxide dismutase [114], catalase [115], glutathione peroxidase, and glutathione reductase [116] are found compared with a healthy control group. A similar pattern has been shown in breast [117] and lung [118,119] carcinoma, progressive bladder carcinoma [120], and lymphocytic leukemia [121,122]. It should be noted that the observed changes in the activity of enzymes are significantly worsened as disease progresses, which indicates their role in the pathogenesis of malignant neoplasms. As for GSH, the levels of this antioxidant play a diametrically opposite role in the development of cancer diseases [123]. Because glutathione, in addition to antioxidant protection, plays a role in many metabolic processes, increasing the sensitivity of the GSH system in response to redox changes in cancer does not have a positive effect. On the contrary, it protects transformed cells from death in a stressful microenvironment, contributing to proliferation, metastatic activity, and the acquisition of resistance to the action of therapeutic agents [124,125,126]. In particular, direct correlations between high levels of the reduced form of glutathione and unfavorable prognostic signs were found in carcinoma of the ovaries [127], breast [128], colon [129], and lung [130], along with leukemia [131] and a number of other types of malignancies.

The attenuation of the antioxidant defense system of the cell is also directly related to the pathogenesis of neurodegenerative disorders [132]. Thus, the measurement of a wide range of enzymatic and non-enzymatic antioxidants in samples obtained from patients with Alzheimer-type dementia demonstrated a decrease in their activity, with a positive correlation as the clinical prognosis worsened [133,134].

### 2.4. ROS-Mediated Signaling Pathways in Oncology and Neurodegeneration

In recent years, the understanding of the role of oxidative stress has expanded significantly, and nowadays it is often considered to be an imbalance that plays a direct role in the regulation of gene expression and related signaling pathways. As important physiological modulators of intracellular signaling pathways, reactive oxygen species are involved in the progression of malignancies and neurodegenerative disorders through the regulation of mitogen-activated protein kinase (MAPK) and phosphoinositide 3-kinase (PI3K)/Akt, the activation of nuclear factor of activated B cells (κB (NF-κB)), and mutations in transcription factors.

1. Mitogen-activated protein kinase (MAPK) are serine–threonine kinase that mediate both the extra- and intracellular signaling that regulates all aspects of cellular functions, including proliferation, differentiation, survival, death, and transformation [135]. MAPK pathways are represented by the three-level kinase cascade, which, when active, phosphorylates various substrate proteins [136]. One of the key signals in the response to which the pathological MARK response occurs is oxidative stress [137], which causes point mutations [18].

To date, the important role of MAPK signaling pathways in the emergence and progression of many diseases has been convincingly proven [138,139], with the focus in recent years on the RAS/RAF/MEK/ERK pathway [140], modifications of which have been proven in almost 50% of cases of human cancers [141]. Thus, the transmission of signals along this pathway and the phosphorylation of the corresponding proteins contribute to the migration and survival of tumor cells [142,143], the degradation of extracellular matrix proteins, and subsequent tumor invasion [144]. Thus, the RAS/RAF/MEK/ERK signaling pathway is considered an important therapeutic target for the development of anti-neoplastic drugs. The significance of other MAPK pathways in the development of malignancies is ambiguous, but, in the case of neurodegenerative diseases, reactive oxygen species (mainly hydroxyl radicals, superoxide anion radicals, and hydrogen peroxide) are typical activators of the JNK and p38 pathways that mediate the downstream negative regulation of β- and γ-secretase activity and the phosphorylation of amyloid precursor protein and tau protein, leading to neuronal death.

2. PI3K/AKT (the signaling pathway of phosphatidylinositol-3-kinase/protein kinase B) is the most important coordinator of intracellular signaling in response to extracellular stimulants, which are free radicals. This signaling pathway plays a central role in the perception of metabolic changes in the environment [145]. Its role is evolutionarily related to the regulation and support of cell growth, proliferation, and survival. Hyperactivation of the PI3K/AKT signaling cascades is one of the most common disorders in cancer [146,147,148], while neurodegenerative disorders are characterized by impaired signaling in this pathway [149,150].

Studies have shown that, in cancers, the PI3K/Akt signaling pathway affects the cell cycle by phosphorylating cyclin-dependent kinase inhibitors and preventing translocation to the nucleus of the tumor suppressor gene p27, thereby attenuating its inhibitory effect on the cell cycle and directly promoting tumor cell proliferation [151]. The activation of this signaling pathway also determines the resistance of transformed cells to apoptotic death by inhibiting the pro-apoptotic factors Bad and procaspase-9 [152], as well as resistance to antitumor therapy in various types of malignant neoplasms, including carcinomas of the prostate [153,154], lung [155,156], breast [146,157,158], esophagus [159], glioma [160], etc.

In the context of the role of PI3K/AKT in the progression of neurodegenerative diseases, it should be noted that, in the brain, this signaling pathway performs a wide range of functions, including complex processes such as dendrite and axon elongation [161,162]. This gives PI3K/AKT a unique role in the maintenance of synaptic plasticity and has a significant impact on the processes of memory formation [163,164,165]. PI3K-Akt disorder, found in the brain in neurodegenerative diseases, provokes mitochondrial dysfunction, which leads to a surge in the generation of reactive oxygen species and the further development of pathological mechanisms [166,167]. In particular, it has been shown that inactivation of the PI3K/AKT pathway correlates with an increase in the level of hyperphosphorylated tau protein [168] and Aβ_40-42_ plaques in the brain [169], mainly due to the increased activity of glycogen synthase-3β (GSK-3β) [168]. To date, it has been documented that antioxidant therapy targeting this signaling pathway is a promising strategy for the treatment of Alzheimer’s disease [166,168,170].

3. Activated B cell nuclear factor (NF-κB) comprises a family of redox-sensitive transcription factors that regulate the expression of various genes [171] and are involved in inflammatory processes [172,173]. NF-κB is a well-known oxidative stress “sensor” that detects H_2_O_2_ at low levels. Under normal conditions, this factor is in a “rest” condition due to its association with the inhibitor of κB proteins (IκB) [174] while inducing stimuli, including free radicals, to trigger the activation of the IκB kinase complex. Thus, in an in vitro model of neurotoxicity, it was found that the treatment of a human neuroblastoma cell culture with H_2_O_2_ promoted the translocation of NF-κB into the nucleus and the subsequent transcription of pro-inflammatory cytokines and chemokines [175].

In recent years, NF-κB has been increasingly recognized as a key factor in all stages of tumor initiation and progression, both as an independent unit [171,176] and in cross-interactions with a variety of other signaling molecules [177]. Free-radical-induced oncogenic mutations leading to NF-κB activation have been identified in various types of malignancies and are associated with a negative prognosis. For example, in the cells of squamous cell carcinoma of the oral cavity, a decrease in the activity of the antioxidant superoxide dismutase and the increased production of ROS correlate with high activity levels of NF-κB [178]. It has been shown that, under the conditions of tumor development, NF-κB has a selective effect in transformed cells: it triggers the activation of survival genes and genes in the tumor microenvironment, contributing to inflammation. It is also of interest that, in neurodegenerative diseases, NF-κB exerts negative effects, not only by inducing neuroinflammation [179,180] but also by stimulating amyloidogenic cascades due to the presence of sites of synthesis in the promoter region of genes involved in amyloidogenesis [181]. All of this evidence suggests that there is a close correlation between this transcription factor and the pathogenesis of cancer and Alzheimer’s disease.

4. Nuclear factor erythroid 2-related factor 2 (Nrf2) is a transcription factor that is considered one of the main coordinators of the cellular antioxidant response. Traditionally, the activation of Nrf2 has been associated with oncological diseases that increase the production of antioxidant proteins and maintain redox balance in tumor cells [182]. However, recent studies have shown numerous functions of Nrf2 that lie beyond its original purpose, opening up the possibility of targeting this factor in the treatment of other diseases, including Alzheimer’s disease [183].

Under normal conditions, basal levels of Nrf2 are maintained at a low level, but when pathological conditions occur, it is activated, accompanied by a rapid suppression of reactive oxygen species and the restoration of oxidative damage through the expression of target genes [184]. This positive feature of this protein in the treatment of various diseases has been discussed in detail since the last century in a number of works, and it had no competition until 2006. Researchers have begun to actively highlight the role of Nrf2 activation, mainly in transformed cells, in the progression of malignant neoplasms, contributing to tumor metastasis [185,186] and the acquisition of resistance to therapy [187] by maintaining reprogrammed cell metabolism under hypoxia [188]. This phenomenon has been described as the “dark side” of Nrf2 [189], which has allowed researchers to make significant progress in understanding the role of the transcription factor in the pathogenesis of cancers.

As mentioned above, a growing body of evidence suggests that altered Nrf2 expression is significantly associated with neurodegenerative diseases, including Alzheimer’s disease [190]. However, in this case, a decrease in the expression of Nrf2 and protein-controlled genes is associated with an increased risk of the development and early onset of Alzheimer’s disease [191]. Thus, in animal models with this cognitive disorder, it was found that, under the conditions of redox imbalance in the brain, the Nrf2-mediated antioxidant response is suppressed, correlating with higher levels of the insoluble form of hyperphosphorylated tau protein [192] and β-amyloid [193]. In addition, in the work conducted by Branca et al., it was shown that Nrf2 deficiency significantly aggravates the cognitive dysfunctions of transgenic APP/PS 1 animals in a study of various types of memory, including spatial, working, and associative, which was associated, in particular, with an increase in the level of Aβ [194]. In turn, the induction of Nrf2 expression can promote the excretion of the precursor protein β-amyloid and tau protein by influencing the downstream genes involved in the processes of autophagy and macroautophagy [195].

All of these data indicate that the impaired expression of Nrf2 as a result of redox imbalance can be considered an important therapeutic target in the search for promising therapeutic agents for the treatment of both malignant neoplasms and Alzheimer’s disease.

**Figure 1:**
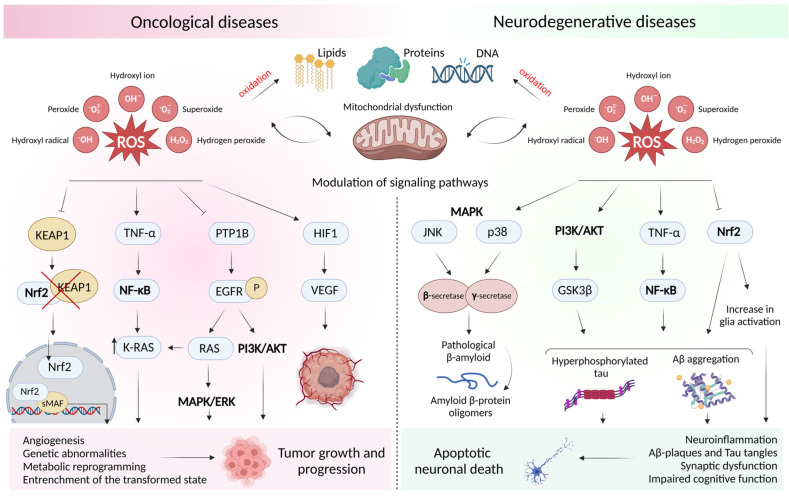
Schematic illustration of the negative role of mitochondrial dysfunction and reactive oxygen species in the development and progression of oncological and neurodegenerative diseases, with an emphasis on the cross-effects of signaling pathways associated with oxidative stress. This figure was created by the authors using BioRender.com (https://www.biorender.com/ (accessed on 14 June 2023)).

Thus, oxidative stress is certainly a significant common denominator that is shared by cancer and neurodegenerative disorders (Figure 1). Although this process is not the only factor in their etiopathogenesis, it creates interesting opportunities for the development of new strategies for the treatment of these socially significant diseases.

### 2.5. Potential Neuroprotective and Antitumor Therapeutic Candidates Targeting ROS

Guided by the fact that most of the known therapeutic agents are directly or indirectly derived from natural resources (more than 70% of the currently existing anti-tumor agents are natural products or their derivatives [196]), we focused on substances of natural origin with high pharmacological potential.

A prominent representative of a natural substance with good potential in the treatment of a wide range of diseases is quercetin (3, 3′, 4′, 5, 7-pentahydroxyflavone), which is a polyphenolic compound.

Among the anti-tumor effects of quercetin is its ability to to induce cell cycle arrest in G2/M and G1 phases through the regulation of the PI3K/Akt [197] and MAPK signaling pathways [198], inhibit proliferation, and trigger cascades of apoptotic tumor cell death [199], as well as reverse resistance to the action of cytostatic agents [200]. These abilities are primarily due to the bioflavonoid’s ability to modulate levels of reactive oxygen species [201]. In particular, in the work of Lu et al. [197], quercetin demonstrated the ability to effectively inhibit the PI3K/Akt signaling pathway in docetaxel-resistant prostate cancer cells LNCaP/R and PC-3/R and, in so doing, to restore the sensitivity of transformed cells to the action of cytostatic agents. This effect also correlated with reduced tumor growth in mice with a xenograft model [197].

This effect was also found in the results of investigations into the chemosensitizing ability of quercetin in ovarian adenocarcinoma [202], where flavonoids, regulating the PI3 signaling pathway K/Akt/mTOR and inhibiting Nrf 2 expression, reversed the resistance of the SKOV-3/CDDP cell line to the action of cisplatin. The sequential treatment of PC3 and DU145 prostate carcinoma cells with vitamin C and quercetin led to a significant decrease in Nrf2 expression and in the activity of glutathione enzymes, which were accompanied by the lower production of endogenous ROS and remarkable levels of cell death [203]. Similar results were obtained in [204] in a study of the therapeutic potential of such a combination against the human breast cancer cells MDA-MB 231. Another mechanism of anti-tumor action of quercetin is its anti-inflammatory function [205], and Lin et al. [206] demonstrated quercetin’s ability to restore the number of leukocytes and reduce the expression of markers of oxidative stress in mice with a model of colorectal carcinoma, which was accompanied by a decrease in the number and size of colon tumors.

The neuroprotective effects of quercetin, associated with the implementation of its antioxidant properties, have also been widely studied [207]. In particular, in the work of Rishitha et al. [208], due to the inhibition of lipid peroxidation and the modulation of glutathione levels, solid lipid nanoparticles of quercetin effectively inhibited Danio rerio cognitive dysfunction with pentylenetetrazole-induced neurodegeneration. The neuroprotective action of quercetin has also been confirmed in a lipopolysaccharide-induced (LPS) model, where the chronic administration of flavonoids to 18-month-old mice with LPS-induced dementia led to significant improvements in memory performance [209]. While evaluating the cognitive function of rats with a model of Alzheimer’s disease stimulated by toxic forms of Aβ1-42, Li et al. [210] also identified quercetin’s positive effects in the Morris water maze test. This was directly correlated with Nrf 2 activation, altered levels of oxidative stress markers, decreased MDA, and the increased expressions of superoxide dismutase, catalase, and glutathione, which ultimately inhibited neuronal damage. Moreover, the three-month administration of the quercetin glycoside quercetrin to transgenic 5xFAD mice led to the leveling of cognitive disorders mediated by the previously mentioned anti-inflammatory function [211]. As such, by modulating transcription factor NF-κB, which is overactivated in neurodegeneration [212], quercitrin interfered with the excessive secretion of inflammatory cytokines, blocking microglial proliferation.

Among natural compounds, sesquiterpene lactones also play an important role in the development of therapeutic agents due to their wide range of biological activities and promising pharmacological profiles [213,214,215,216,217,218]. One of the outstanding discoveries of traditional Chinese medicine is artemisinin, which exhibits excellent anti-malarial activity and is isolated from *Artemisia annua* L. [219,220]. Almost 50 years have passed since its introduction into clinical practice as a priority therapy for tropical malaria, while the range of pharmacological properties exhibited by artemisinin is being continuously supplemented [221].

In many studies, artemisinin and its derivatives have been found to exert anti-tumor effects by inducing oxidative stress. In particular, ROS-dependent cytotoxicity has been shown for water-soluble artemisinin–artesunate in cell models of colorectal carcinoma [222], ovarian cancer [223], non-small-cell lung cancer [224], etc. Interestingly, the anti-tumor effects of artesunate are not limited to an increase in the levels of free radicals, and their toxic effects are strictly selective to cells of tumorous origin [225]. As an inhibitor of NF-κB, artesunate reverses the resistance of metastatic cells of castration-resistant prostate cancer to androgen receptor antagonists, leading to their ubiquitin-mediated degradation [226].

Due to its pronounced antioxidant properties in cells of non-tumorous origin and its ability to penetrate the blood–brain barrier without serious adverse effects, artemisinin has been actively considered by researchers for use as a promising neuroprotective agent. Thus, in a study of the protective properties of artemisinin in Parkinson’s disease induced by the MPP^+^ cell model, it was found that sesquiterpene lactone may inhibit the apoptotic death of SH-SY5Y cells by reducing oxidative damage due to the increase in the activity of the endogenous antioxidant enzymes superoxide dismutase and glutathione and the suppression of levels of malondialdehyde [227]. In an in vivo model of this neurodegenerative disorder, artemisinin reduced damage to dopaminergic neurons in mice treated with the neurotoxin 1-methyl-4-phenyl-1,2,3,6-tetrahydropyridine [228]. The neuroprotective properties of artemisinin have also been shown in the treatment of the HT-22 neuronal cell line with glutamate-induced neurotoxicity, which manifested as the blocking of the production of reactive oxygen species and the activation of the Akt signaling pathway, and, as a result, increased cell survival [229]. In a study conducted by Okorji et al. [230], the authors described the possible therapeutic neuroprotective effect of the fat-soluble artemisinin derivative artemether. Artemether led to the activation of Nrf2 and its binding to elements of the antioxidant response in LPS-stimulated microglia BV2, which directly correlated with a decrease in the levels of inflammatory mediators (prostaglandin E, microsomal prostaglandin E-synthase 1, cyclooxygenase 2, TNF-α, and interleukin 6), as well as in the content of Aβ and the activity of β-secretase 1. Zhao et al. [231] also found that, due to the reduction in oxidative stress and its anti-inflammatory effects, artemisinin has the ability to improve the cognitive functions of triple-transgenic 3xTg cells in the APPSwe, TauP301L, and PS1M146V genes, precisely reproducing the disorders associated with Alzheimer’s disease.

Other representatives of the group of sesquiterpene lactones also exhibit potent therapeutic properties due to the modulation of the redox balance: costunolide, first obtained from the roots of *Saussurea lappa*, C.B. Clarke, and parthenolide, extracted from *Tanacetum parthenium* L. The decreased viability of human bladder cancer cells under the action of costunolide is associated with the hyperproduction of reactive oxygen species and the disruption of the transmembrane potential of mitochondria, leading to the overexpression of apoptotic proteins, the suppression of tumor suppressors and, ultimately, the triggering of an apoptotic cascade of cell death [232]. Similar effects of costunolide were demonstrated by Hua et al. [233] in a study of the anti-tumor properties of costunolide in a model of human esophageal squamous cell carcinoma, as well as breast ductal adenocarcinoma [234]. In turn, study of the therapeutic potential of parthenolide has shown that it may induce the death of cervical cancer cells by blocking the PI3K/Akt signaling pathway and inducing intensive ROS formation, leading to the dissipation of mitochondrial membrane potential [235]. The possibilities of the administration of parthenolide in the treatment of triple-negative breast cancer have been demonstrated in a study [236]. Thus, the generation of ROS caused by this compound in the MDA-MB231 cell line led to the depletion of the reduced form of glutathione and the shutdown of transcription factor NF-kB, with subsequent cell death. Moreover, recent work carried out by the research team led by Jorge studied the antitumor activity of parthenolide in lymphoid neoplasms: multiple myeloma, diffuse large-B-cell lymphoma, T- and B-cell acute lymphoblastic leukemia, and Burkitt’s lymphoma [237]. Interestingly, the mechanisms of the antitumor action of parthenolide differed depending on the cell line, but, in all cases, sesquiterpene lactone promoted cell death along the apoptosis pathway due to a significant increase in ROS and a decrease in GSH activity.

Similar to artemisinin, costunolide and parthenolide are also actively being considered as potential neuroprotective agents. Treatment with costunolide of the PC12 cell line obtained from the pheochromocytoma of rat adrenal medulla prevented damage to cells with neurotoxin H_2_O_2_ [238]. Due to a decrease in the level of intracellular reactive oxygen species, costunolide reduced the expression of caspase 3, which is involved in the apoptosis process. Similar neuroprotective properties were found for parthenolide. In a model of transgenic APP/PS1 mice, parthenolide significantly improved memory performance in the Morris water maze test, perhaps due to its antioxidant properties and ability to reduce neuroinflammation by blocking the AKT/MAPK/NF-κB signaling pathway [239]. Moreover, in the work of Arslan et al. [240], the ability of costunolide and parthenolide to inhibit the activity of the enzyme monoamine oxidase B was considered a possible mechanism of neuroprotective activity in a cellular model of Parkinson’s disease.

Table 1 presents the key features of the chemical compounds described above, allowing them to be considered as medicinal agents capable of influencing processes associated with oxidative stress.

## 3. General Aspects of the Epigenetic Regulation of Neurodegenerative Diseases and Cancer Pathogenesis

### 3.1. Histone Deacetylases as Major Epigenetic Regulators: Structure and Function

It is well known that the structure of chromatin is made up of DNA and histones. In total, 146 pairs of DNA bases are tightly wrapped around an octamer of histone proteins, including two copies of H2A, H2B, H3, and H4, with H1 being a linker histone protein [241,242]. The long N-terminal regions of histone proteins undergo many post-translational modifications, including acetylation, methylation, phosphorylation, sumoylation, ubiquitination, etc. [243,244,245].

**Figure 2:**
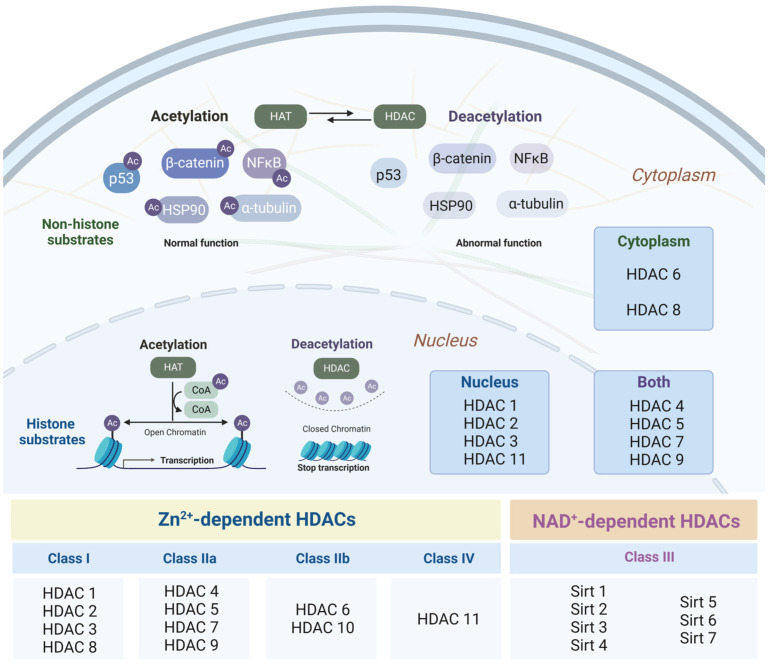
Histone acetylation and deacetylation processes as fundamental mechanisms of epigenetic regulation that controls gene expression. Acetylation is mediated by the activity of histone acetyltransferase enzymes (HATs), which catalyze the attachment of acetyl groups to the lysine residues of histone proteins, which contributes to the relaxation of chromatin conformation and the triggering of transcription. Histone deacetylases, on the contrary, remove acetyl groups from histones, which leads to a thickening of the chromatin structure and the suppression of gene transcription. Histone deacetylases also implement their functions through modifications of non-histone substrates. This figure was created by the authors using BioRender.com https://www.biorender.com/ (accessed on 20 June 2023).

Histone acetylation and deacetylation are the most important epigenetic processes that influence chromatin status and gene expression. Representing tightly regulated dynamic processes, they are controlled by a fluctuating balance between the reversible activity of enzymes of two families: histone acetyltransferases (HATs) and histone deacetylases (HDACs) (Figure 2) [246,247,248].

The acetylation process consists of adding an acetyl group to the N-terminal lysine of substrates, which leads to a decrease in the positive charge of histone proteins to neutral and prevents them from binding to negatively charged DNA [246,249]. This formation of the loose structure of chromatin, euchromatin, is associated with transcription activation. In turn, deacetylation, the reverse process of acetylation, involves the removal of the acetyl group from lysine residues in the tails of proteins, which enhances the interaction between positively charged histones and negatively charged DNA [250]. This strong binding of DNA to histone proteins contributes to the formation of a permissive state of chromatin, which interferes with gene transcription. Interestingly, as enzymes that limit this process, histone deacetylases also have the ability to regulate other post-translational modifications, such as methylation, ubiquitination, and sumoylation [251]. For example, acetylation has been shown to inhibit proteasome-mediated protein degradation, dependent on ubiquitination [252]. All of this has led to a genuine interest in the role of HDACs as critical regulators of the normal and pathological functioning of the body. Despite the fact that the first documented studies of the enzymatic activity of histone deacetylases date back to relatively recent works published in the early 1970s, since then, approximately 15,000 articles have been published on this topic (according to a systematic literature search for original articles in the PubMed database); as a result, important discoveries have already been conducted in the field of HDAC investigations.

The range of biological functions of HDACs includes cell proliferation [253] and differentiation [254], inflammatory responses [255,256], DNA damage [257], and apoptosis [256]. In addition to their role in transcriptional repression, HDACs also act as modulators of non-histone post-translational modifications of proteins of various natures, including transcription factors and signaling mediators [258]; therefore, histone deacetylases potentially play a role in almost every aspect of the body’s functioning. As a result, HDACs have been closely studied by researchers in therapeutic experimental paradigms [253]. Among the many roles that HDACs play in human diseases, oncological diseases are the most frequently discussed.

### 3.2. Changes in the Intensity of Histone Acetylation during Oncogenesis

The aberrant activity of histone deacetylases is often associated with tumor progression; as a result, enzymes of this class have been recognized for more than 30 years as key targets for the action of therapeutic agents against various types of malignant neoplasms [258,259,260].

Numerous reports state that the overexpression of HDACs is observed in both solid tumors and hematologic malignancies, a finding that correlates with multiple clinical and pathological parameters and low patient survival.

According to preclinical and clinical studies, it is class I HDAC that contributes to the development of malignant neoplasms.

Thus, HDAC1 is an important epigenetic factor in lung carcinoma [261], and there is a close correlation between its expression and the degree of histological differentiation, as well as the subtype [261]. HDAC1 expression was found to be higher in squamous cell carcinoma than in lung adenocarcinoma [262]. The level of HDAC1 expression can also serve as a good diagnostic and prognostic marker of malignant neoplasms of the gastrointestinal tract [261], especially in colorectal carcinoma [263,264]. When analyzing the relationship between HDAC1 expression and clinical features, it was found that the activity of this isoform of histone deacetylase was higher (1) in patients with stages III–IV than in patients with stages I–II of gastric cancer [265,266,267] and liver cancer [268]; (2) in patients with poorly differentiated liver cancer [269] and adenocarcinoma of the large intestine than in patients with medium- and high-grade cancers [270]; and (3) in groups of persons with positive lymph node metastasis in gastric [265,271,272] and hepatic [268] carcinomas and distant metastases in colorectal carcinoma [263]. A recent study of the possible mechanism of action of histone deacetylase 1, conducted by Yu et al. [273], demonstrated that HDAC1 is involved in the stimulation of the proliferation of malignant gastric cells by enhancing the expression of long non-coding RNAs that regulate the activity of both oncogenes and tumor suppressors [274,275,276,277]. Moreover, HDAC 1 has been found to play an integral role in tumor cell evasion from the immune response due to the γ-interferon-induced expression of homologue B7 1 (B7-H1), which plays a fundamental role in the initiation and progression of gastric carcinoma [278]. At the same time, in the study conducted by Jiang et al. [279], the analysis of primary tumor samples obtained from patients with gastric cancer showed that the overexpression of HDAC1 is accompanied by a high value of maximum standardized uptake and a poor prognosis. This is due to the direct effect of the enzyme on the activity of the factor induced by hypoxia 1-alpha (HIF-1α), leading to a shift in the metabolic state of tumor cells towards glycolysis.

Epigenetic modifications associated with the overexpression of HDAC1 also play a causal role in the progression of breast cancer (BC). Guo et al. [280] have shown that high levels of the expression of histone deacetylase 1 correlate with clinical and pathological signs and a negative prognosis in patients with breast cancer, with a direct relationship between HDAC 1 levels and the histone binding protein RBBP4 responsible for tumor cell invasion and migration. HDAC1 levels in the cells of this malignancy are higher than those in normal cells; this contributes to their proliferation and migration by regulating the transcriptional and promotional activity of interleukin 8 [281], which plays a significant role in numerous oncogenic pathways [282]. At the same time, in a recent review by Sukocheva et al. [283], the defining role of epigenetic regulation in breast cancer resistance by estrogenic receptor modulators was noted. Thus, the abnormal expression profile of HDAC1 acts as an enhancer for the development of multidrug resistance (MDR) in breast cancer cells, leading to the blocking of estrogen receptors along with the induction of tumor activator genes. A similar effect may also be related to the ability of HDAC1 to enhance the expression of P-glycoprotein (P-gp), a membrane protein that is a critical transporter of drug efflux [284], by recruiting transcription coactivator P300/CBP-associated factor (PCAF) and nuclear transcription factor Y (NF-Y) subunit α to the P-gp promoter region [285]. The recent studies by Duan et al. [286,287] were the first to investigate the role of HDAC1 in the regulation of another protein with similar P-gp functions: placental breast cancer resistance protein (BCRP), which is overexpressed in tumor cells. It was found that histone deacetylase 1 activity has a positive correlation with BCRP expression in placental breast cancer cells.

Increased HDAC1 activity has also been found in prostate cancer [288,289]. In particular, in a study conducted by Halkidou et al. [290], an immunohistochemical analysis of HDAC1 expression was performed in samples obtained from malignant prostate lesions in humans and mice with a CWR 22 xenograft model. The significant activation of this enzyme produced an aggressive, strongly proliferating phenotype and the metastatic potential of cells mediated by the suppression of the activity of the tumor suppressor p53 and inhibitors of the cyclin-dependent kinases p 21 and p27. A pronounced prognostic effect of HDAC1 on prostate cancer was shown in a large-scale analysis of samples in a study conducted by Burdelski et al. [288], where nuclear accumulation HDAC 1 was closely correlated with tumor aggressivity and poor prognosis, as was also confirmed in earlier studies [291,292]. Considering one of the possible mechanisms of oncogenic action of histone deacetylase 1, Shankar et al. [293] identified the HDAC1-mediated repression of Maspin, a tumor-suppressor gene that regulates cell invasion, angiogenesis, and apoptosis, processes important for both tumor growth and metastasis [294,295].

Histone deacetylase 1 is a predictor of an unfavorable tumor phenotype in gynecological cancers: ovarian carcinomas [296] and cervical carcinomas [297]. A global study of the relationship between the immunohistochemical expression of HDAC1 and the clinical and pathological data of patients with ovarian cancer revealed the maximum increase in nuclear expression in the samples obtained in patients with mucinous carcinoma, 80% with clear cell carcinoma, more than 70% with serous carcinoma, and 53% with endometrioid carcinoma [298], indicating a clear correlation of HDAC 1 levels with the prognosis. A study on chemoresistant A2780-AD ovarian cancer cells of the molecular mechanisms of epigenetic modifications associated with HDAC1 overexpression showed the mediated HDAC1 suppression of the G-protein 10 signaling regulator (RGS10), which performs a key function in inflammation and cell survival [299]. A number of studies have shown that the deficiency of this protein is associated with the development of resistance to therapy in transformed cells by increasing the production of TNF-α and cyclooxygenase 2 (COX-2), which mediates the production of prostaglandin E2 (PGE) 2) [300,301]. Two years later, the same team of authors, led by Cacan [302], found that an increase in the activity of HDAC 1 in the A2780-AD cell line also accompanied a decrease in the level of acetylated histone 3 (H3) in the protective region of the FAS antigen, as a result, blocking its receptor function, which consists of the induction of programmed apoptotic cell death. In the work by Liu et al. [303], HDAC1 activation was also shown to be an important event in the development of drug resistance, with the results indicating that the possible mechanism of this enzyme’s action was of particular interest. The team of authors found that the abnormal expression of HDAC1 in cisplatin-resistant ovarian cancer cells stimulates cell proliferation and chemoresistance by regulating the c-Myc-miR-34a pathway, which stimulates the aberrant expression of the transcription factor c-Myc and suppresses the activity of the powerful miR-34, a tumor suppressor, two known regulators of multidrug resistance [304,305,306,307].

As for cervical cancer, Liu et al. [297] found that the aberrant activity of HDAC1 in C-33A cells significantly increases the expression of octamer-binding embryonic transcription factor 4 (Oct4), a prognostic biomarker of various types of malignancies that plays a critical role in maintaining the pluripotency and self-renewal of embryonic stem cells [308,309,310,311]. The contribution of histone deacetylase 1 to the maintenance of stem properties in transformed cells was also shown in a recent study by Yokoi et al. [312], where an abnormal HDAC 1 expression profile in the ME180 and CaSki cell lines led to the activation of the Oct4, Nanog, and SOX2 genes that exhibit oncogenic function.

Changes in the activity of other isoforms of class I HDACs may also have clinical value as therapeutic targets. However, nowadays, the understanding of their role in the pathogenesis of cancer is at fairly early stages compared to the understanding of HDAC1.

However, it has been thoroughly proven that HDAC2 is overexpressed in tissue samples obtained from patients with lung cancer, with a negative correlation between HDAC2 levels and prognosis [313]. Such a function of histone deacetylase 2 in the processes of the migration and invasion of lung cancer cells can be implemented by regulating the expression of protein complexes, such as eukaryotic initiation factors 5 and 6 (eIF5 and eIF6), which play a decisive role in the occurrence and progression of tumors [314,315,316,317]. The ability of HDAC 2 to control the metastasis of transformed cells may also be mediated by other mechanisms. In [318], an increase in HDAC 2 levels in non-small-cell lung cancer (NSCLC) cells led to an increase in the expression of fibronectin, a protein that is an extracellular driver of malignancies [319,320,321]. Wang et al. have shown that HDAC2 activates the expression of c-Myc and cyclin D1, which promote the proliferation, migration, and invasion of NSCLC cells [322]. The expression levels of histone deacetylase 2 are also impaired in colorectal cancer [323]. The analysis of specimens obtained from patients with colorectal carcinoma showed a significant increase in HDAC2 and abnormal levels of H3K56 acetylation in tissues [263]. Clinical and pathological data presented in previous work [324] showed that a higher expression of HDAC 2 correlates with poor overall survival and is associated with liver metastasis. Histone deacetylase 2 levels are also associated with tumor aggressiveness in gastric cancer [325]. Thus, there is a statistically significant increase in HDAC2 expression as the disease progresses, along with positive metastasis to the lymph nodes. HDAC2 may also be a potential biomarker for the prognosis of tumor progression in squamous cell carcinoma of the oral cavity [326], esophageal squamous cell carcinoma [327], gallbladder carcinoma [328], and breast carcinoma [329].

As a key component involved in DNA replication and repair and maintenance of the chromatin structure, histone deacetylase 3 plays a number of important roles in regulating cell progression, differentiation, and other processes associated with the progression of malignant neoplasms [330,331,332]. Abnormalities in HDAC expression are often found in the most common types of oncological diseases, in particular, in various histological forms of non-small-cell lung cancer [333,334,335,336], prostate cancer [337], gastrointestinal malignancies [332,338,339,340,341,342,343], glioma [344], etc. Beyer et al. [345] found that the overexpression of HDAC3 mediates the growth of transformed cells in human acute myeloid leukemia by modulating the leukemia-related transcription factors β-catenin, the Wilms tumor suppressor gene (WT1), and the myelocytomatosis oncogene (MYC). The aberrant activity of HDAC3 in colon adenocarcinoma cells, SW480, significantly reduces H4-K12 histone acetylation and modulates gene expression in the intracellular signaling pathway Wnt [346], the hyperactivation of which positively controls a number of key cascades regulating stem properties, metastasis, and immune control in neoplastic cells [347,348,349]. Moreover, it has been found that HDAC3 promotes not only the proliferation and invasion of transformed cells but also the acquisition of drug resistance by suppressing the transcription of tumor suppressor genes p 53, p27, and Bax [350] and activating the PI3K-Akt-mTOR pathway [351].

Another histone deacetylase of class I, the deregulation and overexpression of which are involved in various aspects of the progression of malignant neoplasms, is HDAC8. Despite the fact that this isoform was identified relatively recently as the last representative of class I HDACs, advanced methods allowed researchers to clearly identify the structure and function of the enzyme and determine its significance in oncological diseases [352,353,354]. By acting on both histone and non-histone substrates, overexpressed HDAC8 implements its oncogenic functions through the regulation of a number of signaling cascades in various types of malignant neoplasms, in particular, in hepatocellular carcinoma [355], gastric adenocarcinoma [356], acute lymphoblastic leukemia [357], squamous cell carcinoma of the oral cavity [358], neuroblastoma [359], etc. [360,361,362]. Thus, the study of the molecular mechanisms of the HDAC8-induced proliferation of tumor cells showed that, by reducing the expression of cytokine signaling suppressors 1 and 3 (SOCS1 and SOCS3), histone deacetylase 8 contributes to the constitutive activation of the Janus kinase signaling pathway 2/signal transducers and activators of transcription signaling (JAK2/STAT) [363], which play an important role in the oncogenesis of myeloproliferative neoplasms and leukemia [364]. A recent study by Zhang et al. [365] showed that the deacetylated conserved residue of K62, the key enzyme pyruvate kinase glycolysis M2 (RCM2), facilitates the transport of PKM2 into the nucleus, enhancing its enzymatic activity; it also binds β-catenin, promoting the transcription of the gene encoding cyclin D (CCND1), which stimulates the progression of numerous malignancies [366,367,368] and cell cycle development. HDAC 8 is also involved in the metastasis of many cancers [354,369,370]. Similar to the first isoform of histone deacetylase, HDAC 8 also promotes the migration of prostate carcinoma cells by inhibiting the expression of the tumor suppressor maspin [293], and the functional redundancy of HDAC8 in human cervical cancer cells (HeLa) promotes metastasis through the excessive deacetylation of tubulin [371]. Neoplastic cell migration in triple-negative breast cancer is significantly enhanced by the HDAC8-induced modulation of mesenchymal markers such as matrix metalloproteinases 2 and 9 (MMP-2 and MMP-9), N-cadherin, fibronectin, and vimentin [372].

### 3.3. Role of Histone Deacetylases in the Pathogenesis of Neurodegenerative Disorders

The role of histone deacetylases in the regulation of brain functions, neurological status, and pathogenesis of a wide range of neurodegenerative conditions has been investigated using a huge number of experimental models [373]. It was found that, by reducing the acetylation of histone proteins, as well as non-histone substrates, individual isoforms of histone deacetylases cause the suppression of the transcription of the regulatory genes involved in neuroplasticity, learning, and memory processes, leading to the development and progression of central nervous system disorders.

For 15 year, researchers have focused on the relationship between the sixth isoform of histone deacetylase and Alzheimer’s disease. Because of this, nowadays, HDAC6 has become a widespread therapeutic target for the treatment of this neuropathology [374]. Histone deacetylase 6 is a unique enzyme since it implements its functions through both epigenetic and non-epigenetic mechanisms, regulating a variety of signaling pathways associated with neurodegenerative disorders [375]. In an analysis of the levels of different isoforms of histone deacetylases in the frontal lobe of the brains of patients with mild, moderate, and severe Alzheimer’s disease, a significant increase in HDAC6 was found, which negatively correlated with overall cognitive status as the disease progressed [376]. Meanwhile, in the work of Bai et al., positron emission tomography imaging of the brain of transgenic 5xFAD mice, modeling dementia of the Alzheimer type, showed a significantly higher radioactivity of the probe [^18^F]PB118 for HDAC6 imaging in the cortex and hippocampus, the regions most susceptible to the disorder [377].

Due to the predominantly cytoplasmic localization inherent in histone deacetylase 6, which differentiates this enzyme from other HDACs, the spectrum of specific non-histone substrates and proteins deacetylated by HDAC6 includes tau protein, α-tubulin, ubiquitin, heat shock protein 90, etc. [378,379].

It is known that the level of acetylation of α-tubulin, the main substrate of HDAC6 [378], plays a key role in the formation of stable microtubules, which are important in various biological processes, including learning and memory [380,381]. The overexpression of HDAC 6 leads to intensive deacetylation of α-tubulin, leading to the destabilization of microtubules and, as a result, the pathological death of neuronal cells that contribute to neurodegeneration [382]. Moreover, HDAC6 is a critical regulator of the ubiquitin–proteasome system [383,384], processes responsible for the degradation of tau protein [385,386]. In the brain organelles of transgenic mice overexpressing HDAC6, there are high levels of aggregated forms of phosphorylated tau protein [387]. Additionally, in the work of Balmik et al. [388,389], the excessive interaction of the ubiquitin-binding domain of histone deacetylase 6 with tau led to conformational changes in the protein, as well as a decrease in the trend of disaggregation of already formed aggregates. The correlation of high tau protein concentrations with abnormal HDAC6 levels was proven via the quantification of neurons in the cerebral cortices of mice exhibiting high expression of this enzyme, while neurons knocked out by this protein showed a decrease in the formation of neurofibrillary tangles by approximately 90%, which was accompanied by an increase in viability [390]. A similar clearance of hyperphosphorylated tau protein under the conditions of blocking HDAC6 activity was also shown by Cook [391] and Sreenivasmurthy [392].

The overexpression of HDAC6 also influences the processes associated with autophagy. There are several ways to regulate the HDAC6 process, the most important of which is deacetylation of the autophagy related transcription factors TFEB and FOXO1, which leads to blocking their translocation to the nucleus and the inhibition of transcription associated with gene autophagy. As a result, the processes of the fusion of autophagosomes with lysosomes are disrupted, and there is no degradation of misfolded proteins (tau protein, Aβ, and others) and mitochondria with impaired functions, which ultimately leads to the death of the neuronal cell [393,394,395]. In recent work by Liu et al., it was found that the aberrant activity of HDAC6 is a critical regulator of neuroinflammatory responses, leading to the overexpression of inflammatory cytokines such as interleukins 6 and 1 β (IL-6, IL1β), as well as TNF-α.

In addition, the aberrant activity of HDAC6 has been demonstrated in other neurodegenerative diseases such as Parkinson’s disease [396] and Huntington’s disease [397].

Researchers in the field of Alzheimer’s disease have also focused on other isoforms of histone deacetylases, which are also involved in pathogenesis, though to different degrees. There is a clear correlation between the overexpression of histone deacetylase 2, belonging to class I, and impaired brain function. In particular, in the work of Guan et al. [398], the analysis of promoter occupancy showed that histone deacetylase 2 may negatively regulate the activity of the genes involved in synaptic plasticity and memory formation, while the overexpression of HDAC2 in the brains of genetically modified mice contributed to a significant disruption of these functions. Such a repressor function of HDAC2 was also shown in p25 transgenic animals modeling Alzheimer’s disease [399], in which an increase in the level of this histone deacetylase isoform correlated with cognitive deficits due to the higher recruitment of HDAC2 on the promoters of key genes associated with learning, memory, and neuroplasticity when compared with wild-type animals. Genes affected by HDAC2 include the brain-derived neurotrophic factor and the early growth response protein 1. It is interesting that, despite the 85% structural homology of HDAC2 to HDAC1, it is the second isoform of histone deacetylase that is involved in cognitive processes [398]. However, even though histone deacetylase 2 would seem to represent an appropriate target for the treatment of neurodegenerative disorders, caution is needed in the use of modulators of its activity. This is because the catalytic function of HDAC2 is a critical requirement of brain neurogenesis in adults [400].

Among representatives of Class I, nuclear-localized HDAC3 is also significantly increased in the hippocampus of APPswe/PS1dE9 transgenic animals, which correlates with high levels of Aβ, the activation of microglia, and a decrease in the density of dendritic spines in the brain. This causes the negative regulation of histone deacetylase 3 and spatial memory in the line of mice [401]. In addition, Bardai and d’Mello [402] found that HDAC3 is a protein with prominent neurotoxic activity since the overexpression of this enzyme leads to the selective death of neuronal brain cells without influencing the survival of cell lines of other origins. With regard to HDAC4, Fitzsimons et al. [403] proved its role in the memory processes of the model genetic organism of Drosophila (fruit fly), but it was shown that, depending on the localization, this isoform is not only a repressor of long-term memory, but also modulates its normal formation, which, as was the case for HDAC2, complicates the use of targeted therapy.

For other isoforms of histone deacetylases, specific correlations with Alzheimer’s disease have not been proven to date.

Thus, nowadays, we have a good understanding of 18 HDAC proteins in humans, which function as transcriptional repressors and corepressors, leading to genuine interest in enzymes of this class. Despite the fact that information about the structural and functional features of histone deacetylases is increasing exponentially, there are still many questions regarding how particular isoforms of HDACs regulate signaling cascades and gene activity, which largely remain unexplored. Nevertheless, there is indisputable evidence that enzymes of this class are involved in the pathogenesis of a wide range of diseases, mainly malignant neoplasms and neurodegenerative disorders, which lays the theoretical foundation for the clinical use of HDACs as targets for the action of promising pharmacological agents.

**Figure 3:**
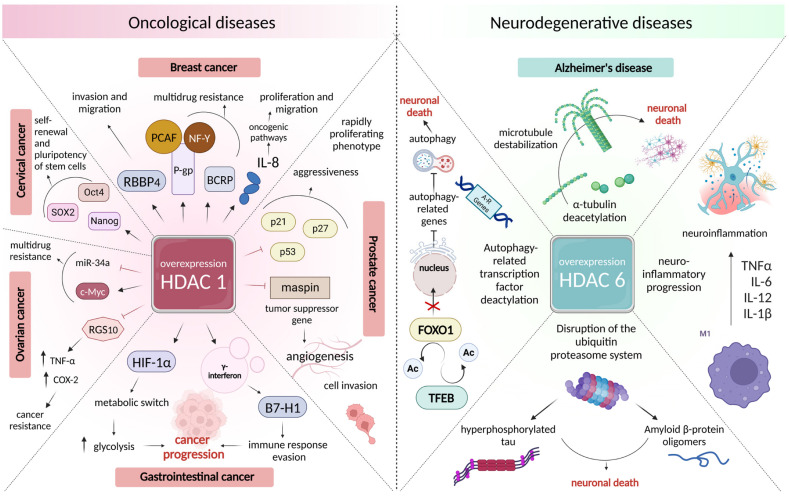
Schematic representation of the pathological functions of HDAC1 in various types of malignant neoplasms and HDAC6 in Alzheimer’s neurodegeneration. HDAC1 overexpression promotes oncogenesis by enhancing the proliferation of tumor cells and suppressing apoptosis, as well as metastasis and the development of drug resistance. In turn, aberrant HDAC6 levels are involved in the pathogenesis of neurodegenerative diseases, leading to the destabilization of microtubules and participating in the processes of neuroinflammation, as well as causing disorders in the ubiquitin–proteasomal system and autophagy processes, preventing the degradation of improperly folded proteins. This figure was created by the authors using BioRender.com (https://www.biorender.com/ (accessed on 17 June 2023)).

In Figure 3, we combine the most important information about the mechanisms of action of the most common histone deacetylases, HDAC 1 and HDAC6, as key epigenetic regulators of the pathogenesis of onco- and neurodegenerative diseases.

### 3.4. Advances in the Development of Histone Deacetylase Inhibitors in the Treatment of Cancer and Neurodegenerative Diseases

**Figure 4:**
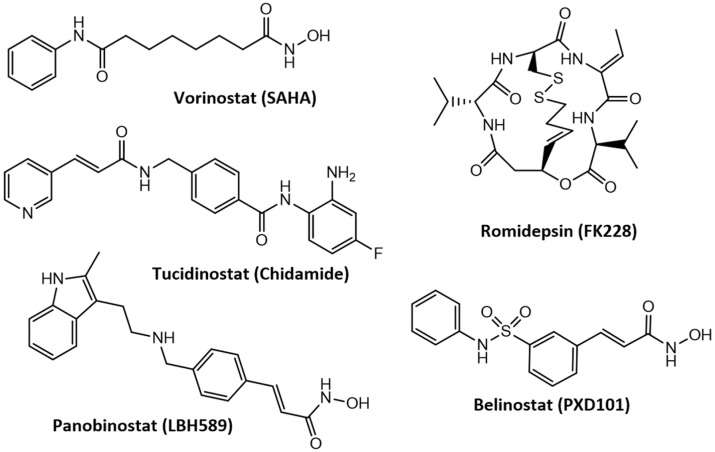
FDA-approved histone deacetylase inhibitors.

Currently, the Food and Drug Administration (FDA) has approved the use of five histone deacetylase inhibitors: vorinostat (suberoylanilide hydroxamic acid, SAHA), romidepsin (FK228), tucidinostat (hidamide), belinostat (PXD101), and panobinostat (LBH 589) (Figure 4).

Based on the formulas presented in Figure 4, it is easy to see that HDAC-inhibitory ability has been found for representatives of different chemical structures. According to convention, all of the histone deacetylase inhibitors that currently exist can be divided into four large groups: (1) hydroxamic acids, (2) benzamides, (3) cyclic peptides, and (4) aliphatic fatty acids. Meanwhile, of all the HDAC inhibitors, it is hydroxamic acid-based compounds that represent the largest and most clinically successful class due to the fact that they exhibit the most promising profile of pharmacological activity [404,405,406].

Nowadays, there are many studies on the therapeutic potential of hydroxamic derivatives, and it is not possible to cover all such works in this paper. Moreover, many review manuscripts, including our recently published large-scale review, have already been devoted to this problem [407]. In this regard, in this section, we decided to select an original strategy for considering the pharmacological prospects of one of the already approved representatives of HDAC inhibitors: vorinostat. Our review will provide convincing evidence of the prospects of the hydroxamic acid class as unique therapeutic agents and will not only summarize the well-known antitumor properties for them, but also the relatively recently discovered neuroprotective effect.

Vorinostat was the first histone deacetylase inhibitor approved by the FDA in 2006 as a monotherapy for the treatment of patients with refractory or recurrent cutaneous T-cell lymphoma [408,409]. To date, a large number of preclinical and clinical trials of vorinostat in the treatment of other hematological and solid tumors have been conducted. However, studies of the antitumor properties of vorinostat monotherapy for solid tumors showed insufficiently satisfactory results [410,411]. This prompted the teams of authors to attempt to study the efficacy of vorinostat in combination with other active compounds that are already used in the treatment of various malignant neoplasms. In particular, the combined use of vorinostat and radioligand ^131^I-metaiodobenzylguanidine has demonstrated a high true-response rate in patients with relapsed or refractory neuroblastoma [412]. The inclusion of vorinostat and isotretinoin in the intensive chemotherapy regimen of medulloblastoma allowed researchers to achieve an improvement in the rates of five-year progression-free survival and overall survival in younger children [413]. The administration of SAHA in combination with autophagy targeting hydroxychloroquine in patients with metastatic colorectal cancer showed a high response of antitumor immunity in a randomized phase II trial [414]. This method enhanced the expression of lysosomal protease of cathepsin D, the p62 protein, and, as a result, the inhibition of autophagy and the subsequent apoptosis of transformed cells [415]. The enhancement of the antiangiogenic properties of pazopanib under the action of SAHA was also found in phase I studies of metastatic solid tumors with TP53 mutations [416]. Here, patients treated with vorinostat + pazopanib demonstrated a significantly longer average time of overall survival and progression-free life, which may be associated with the triggering of mutant p53 degradation mechanisms and the suppression of vascular endothelial growth factor (VEGF)-mediated overexpression of HIF-1α. The potential of the strategy of the combined administration of antitumor agents with epigenetic modulators was also confirmed by the results obtained when combining vorinostat with chemoradiotherapy for the treatment of squamous cell carcinoma of the head and neck, where the selected administration regimen demonstrated high efficacy (96.2% of patients showed a positive response) and safety [417], which has also been shown for other cancer types [418,419].

Based on the results obtained in recent years, there is strong evidence of the therapeutic value of vorinostat in diseases affecting the brain [417]. This is because, as a pan-selective inhibitor, vorinostat modulates the activity not only of HDACs of class I, which perform a predominantly oncogenic function, but also of HDAC6, an isoform that plays a leading role in the functioning of neurons. Over the past 10 years, researchers have accumulated an impressive pool of data on the potential of reprofiling the drug agent as a drug for neurodegenerative disorders.

In the work conducted by Chen et al. [420], vorinostat demonstrated a protective effect under the modeling conditions of neurotoxicity induced by lipopolysaccharides and the N-methyl-4-phenylpyridinium cation (MPP^+^), increasing the viability of dopaminergic neurons by inhibiting deacetylation histones and mediating the release of neurotrophic factors from astroglia. It has also been reported that vorinostat exerts neuroprotective effects by stimulating the expression of glycoprotein clustering in human astrocyte cells, which play a known role in modulating Aβ aggregation in Alzheimer’s disease [421]. Kilgore et al. [422] showed that intravenous injections of vorinostat into transgenic APPswe/PS1dE9 mice led to the restoration of contextual memory in the animals, which directly correlated with the inhibition of HDACs of class I and HDAC6. Further in vitro studies of the neuroprotective properties of vorinostat also showed that a decrease in the activity of histone deacetylase as a result of SAHA action was accompanied by improvements in synaptic function. However, in vivo behavioral experiments on a model of transgenic Tg2576 mice failed to confirm the protective effect of the compound, which is associated with its limited availability to the brain and effective peripheral distribution [423]. In this regard, the teams of authors focused on another known property of vorinostat: its adjuvant ability. Thus, in the work of Sarathlal et al. [424], the administration of vorinostat as a chemosensitizing agent, in combination with the hypoglycemic agent rosiglitazone, resulted in the significantly higher gene expression of neurotrophic factors and attenuated biochemical, cellular, and behavioral abnormalities in a mouse model of streptozotocin-induced Alzheimer’s disease. Interestingly, the authors also managed to improve the profile of the therapeutic efficacy and bioavailability of this system by improving the forms of agent administration using a poloxamer-stabilized system of polymer nanocarriers. One year later, the same group of authors also managed to confirm the potential of vorinostat as a means of enhancing the activity of anti-insulin-resistant drugs [425]. The combined administration of vorinostat and rapamycin resulted in the alleviation of cognitive dysfunction in rats with advanced insulin resistance (IR) undergoing the intracerebroventricular injection of Aβ_1-42_. The subsequent analysis of biomarkers associated with neurodegeneration exacerbated by IR showed a significant decrease in amyloid precursor protein (APP) due to the increased expression of Beclin 1, glial-cell-line-derived neurotrophic factors (GDNF), brain-derived neurotrophic factors (BDNF), neuronal growth factors (NGF), and the neuronal markers MAP 2 (microtubule-associated protein 2) and LAMP 2 (lysosome-bound membrane protein 2). The ability to effectively alleviate cognitive deficit symptoms in a mouse model of Alzheimer’s disease has also been shown for the combination of vorinostat with tadalafil. This combination is aimed at another target, phosphodiesterase type 5, which is a critical component of the cyclic guanosine monophosphate/protein kinase G (cGMP/PKG) signaling pathway that regulates nerve cell apoptosis [426]. It is notable that the synergistic effect of these drug agents also manifested as a decrease in amyloid and tau pathologies and as an obstacle to the death of hippocampal neurons.

The strategy of using vorinostat as a chemosensitizing therapeutic agent has a significant advantage: the use of extremely low doses of the drug during treatment. It allows for the prevention of the adverse effects it has on a healthy microenvironment due to the absence of the selectivity of action on a particular isoform, ensuring optimal safety values in chronic use.

Table 2 summarizes the above materials, focusing on the most significant points in the development of medicinal agents for the treatment of oncological and neurodegenerative diseases.

Thus, to date, the treatment of cancer and neurodegenerative disorders (mainly Alzheimer’s disease) represents most indications for the class of drugs targeting HDACs. In particular, based on our assessment of the current status of studies involving vorinostat, the large-scale potential of epigenetic therapy was confirmed by representatives of the class of hydroxamic acids, which have already achieved promising clinical progress.

## 4. Alterations in the Bioenergetic Metabolism of Cells during Oncogenesis and Neurodegeneration

### 4.1. Determination of the Main Metabolic Processes of the Cell and Energy Metabolism

Cellular metabolism involves many interrelated pathways, the main purpose of which is to provide the cell with the energy necessary for its functioning [433]. In general, metabolism can be divided into a number of chemical reactions, which include both synthesis (anabolism, assimilation, plastic metabolism) and the cleavage of complex macromolecules (catabolism, dissimilation, energy metabolism). These processes are closely interrelated with each other. Thus, during anabolism, energy and products formed in dissimilation reactions are used. In turn, the enzymes formed as a result of assimilation reactions are necessary for catabolism.

As the energy that is necessary for maintaining cell functions, the active transport of ions and substrates, the biosynthesis of complex macromolecules, etc., is formed as a result of catabolism reactions (dissimilation, energy metabolism), and this process has been studied by biochemistry specialists for more than 150 years [434]. Such investigations are the key to understanding both normal physiological functions and their role in the pathological conditions of the body.

**Figure 5:**
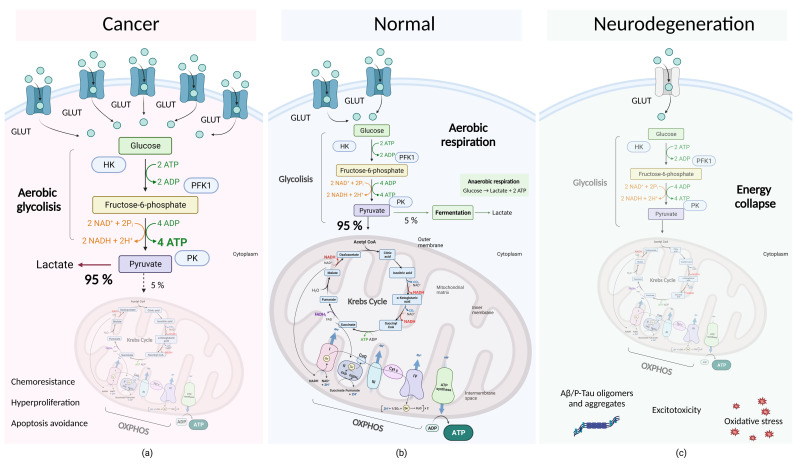
Metabolic reactions occurring in oncological (**a**) and neurodegenerative (**c**) diseases, as well as under the conditions of normal cell functioning (**b**). (**a**) Schematic representation of the biochemical process occurring in tumor cells, which consists of reprogramming the metabolism and the means of obtaining energy from oxidative phosphorylation to glycolysis. (**b**) Illustration of the stages of cellular respiration occurring under physiological conditions. (**c**) Mitochondrial dysfunction and related metabolic disorders that occur in neurodegenerative diseases and lead to an imbalance between energy production and consumption, as well as the hyperproduction of reactive oxygen species. This figure was created by the authors using BioRender.com (https://www.biorender.com/ (accessed on 25 June 2023)).

Under normal conditions, the production of energy by living organisms occurs through the subsequent oxidation of organic compounds during the three main stages of cellular respiration: (1) glycolysis, (2) the tricarboxylic acid cycle, and (3) mitochondrial electron transport (Figure 5).

Figure 5 clearly illustrates disorders in the cellular metabolism in oncological and neurodegenerative diseases, a detailed consideration of which is presented below in the text of the manuscript.

### 4.2. Molecular Subtleties of Tumor Cell Metabolism: Dysregulation of Aerobic Glycolysis and the Warburg Effect

It is well known that tumor cells have a reprogrammed metabolism that promotes their growth, metastasis, and survival [435]. This proliferative metabolic phenotype [436] is characterized by an increased glycolytic function, in which, due to the intensive increase in glucose and its reduction of pyruvate to lactate, transformed cells not only satisfy their high energy requirements but also obtain intermediates that are critical for the synthesis of macromolecules. This phenomenon, often referred to as the “Warburg effect” [437], has been known for about 100 years and is now the object of studies by a large number of scientific groups. Targeting enhanced glycolysis in tumor cells is considered to be a promising strategy for improving the efficacy of the treatment of malignant neoplasms [438].

Until recently, the predominance of the glycolytic pathway in neoplastic cells was a mystery to the scientific community, since, unlike mitochondrial-dependent oxidative phosphorylation (OXPHOS), this pathway is a less efficient way to obtain energy [439], while cells consume a large amount of energy to maintain the tumor phenotype [440,441,442]. However, the results produced in recent years have shed light on the long-standing debate about the significance of this unique phenomenon in tumor cells. Pfeiffer et al. postulated that glycolysis, as a method of producing ATP at a high rate, gives neoplastic cells a selective advantage in the face of competition for common energy sources, thereby giving evolutionary significance to this process [443]. In the study conducted by Locasale et al., the rate of ATP production during glycolysis was recorded as being 100 times higher than that with OXPHOS [444]. In addition, the prevalence of glycolysis may be due to the hypoxic microenvironment of tumors, when mitochondrial defects are observed [445], and oxidative phosphorylation becomes inactive, and obtaining energy as a result of high-rate glucose conversion becomes the only possible way of doing so. For example, mitochondrial dysfunction is observed in malignant neoplasms of the large intestine [446], mammary duct [447], and gastric carcinomas [448].

However, since the Warburg effect is observed even in the presence of fully functional mitochondria, and mitochondrial dysfunction is not necessary for oncogenesis, it has been found that neoplastic cells can acquire a hybrid phenotype using both intensive glycolysis and a standard rate of oxidative phosphorylation (glycolysis/OXPHOS) [449]. It is believed that, due to this phenomenon, tumor cells acquire the property of metabolic plasticity, which plays a particularly important role in metastasis and the development of resistance to therapy. Such properties can be achieved through the glycolysis-dependent activation of the PI3K/AKT signaling pathway, which allows tumor cells to acquire stem properties [450].

In addition to providing cellular energy, metabolic intermediates of glycolysis also play a key role in the biosynthesis of macromolecules, thereby providing a selective advantage to cancer cells under the conditions of a limited nutrient supply. In particular, it has been shown that, by enhancing the hexosamine biosynthesis pathway and the pentose phosphate pathway, aberrant glycolysis can contribute to an increase in the turnover of the nucleotides necessary for the effective repair of DNA damage caused by the action of therapeutic agents [451,452]. In turn, such DNA repairs induce the activation of prooncogenic signaling pathways, triggering the mechanisms of resistance of tumor cells to apoptosis [450,453]. In addition, NADPH and ribose-5-phosphate, formed as a result of the pentose phosphate pathway, are essential for the biosynthesis of lipids and nucleic acids in the tumor, and NADPH also allows neoplastic cells to maintain elevated levels of GSH, which exerts the positive effects for tumor progression described above [454,455].

As shown in Figure 5, which depicts the main biochemical reactions of glycolysis under the action of a number of enzymes, the initial consumption of glucose depends on the specific transporters of GLUT [456], which ensure its transfer from the systemic circulation to the cell [457]. Among the 14 proteins of this family belonging to the major facilitator superfamily of membrane transporters, four isoforms of GLUTs 1–4 play a specific role in the development of glucose, exclusively in glucose homeostasis in cells and in the body as a whole [458,459]. At the same time, it is the overexpression of the representative of GLUT-1 that is observed in many types of malignant neoplasms. In particular, the prognostic value of GLUT-1 expression is indicated in adenocarcinomas of the stomach [460,461], pancreas [462], and prostate [463,464]; carcinomas of the lungs [465] and endometrium [466,467]; and osteosarcoma [468], meningioma [469], and malignant tumors of the oral epithelial tissue [470,471]. In addition, high levels of GLUT-1 expression are found in patients with poorly differentiated breast tumors [472] and are associated with a negative prognosis [473,474,475].

It seems that the blocking of GLUT can be considered a direct approach to glycolysis inhibition as a result of terminating the flow of the substrate into tumor cells, and, as a result, completely disrupting this pathway. However, due to the ubiquitous expression of these transport proteins, this strategy has a critical limitation: the difficulty of developing selective GLUT blockers in neoplastic cells. For this reason, the study of the glycolytic function continues to expand, and, to date, it has been found that the intensity of glycolysis in tumor cells closely correlates with the activity of key glycolytic enzymes, hexokinase, 6-phosphofructo-2-kinase, and pyruvate kinase (Figure 5) [476], which catalyze the irreversible stages of this process; targeting them is thus a promising strategy for the repair of impaired tumor metabolism.

Hexokinase is an enzyme that catalyzes the first stage of glycolysis by converting glucose to glucose-6-phosphate. The limiting role of this enzyme arises due to its kinetic properties, due to which it has an excellent affinity for glucose, which allows the glycolysis process to be triggered even when glucose levels are low, thus playing a key role in the energy metabolism of tumor cells [477]. Among the four existing isoforms of hexokinase in mammals, the contribution of the second isoenzyme (HK 2) to the regulation of the protective functions of neoplastic cells is now well known [478]. In particular, the ectopic expression of hexokinase 2 was found to attenuate apoptosis in a glucose-dependent manner and triggers the Akt-mediated survival of rat fibroblast cell cultures [479]. In addition, a wide range of oncogenic effects of HK2 are shown in hepatocellular carcinoma [480], ovarian malignancy [481,482], non-small-cell lung cancer [483], and prostate [484] and gastric carcinomas [485] and correlates with the recurrence and poor prognosis of breast cancer [486,487].

It has also been shown that, along with an increase in glucose consumption and lactate production, the expression of hexokinase 2, which is found in the prostate carcinoma cell line and has undergone sumoylation, reduces mitochondrial respiration, contributing to the metabolic reprogramming of cells and the triggering of anti-apoptotic mechanisms [488]. Moreover, the oncogenic functions of hexokinase 2 can also be implemented via an alternative non-metabolic pathway. In particular, Wang et al. found a direct correlation between the overexpression of HK 2 and CD133, a key marker of stem cell self-renewal in small-cell lung cancer, due to the glycolytic activity of the enzyme [489]. Similar properties of HK2 have been shown in a model of acute myeloid leukemia (AML). For example, Thomas et al. showed that the interaction of HK2 with nuclear proteins of leukemic stem cells regulates the openness of chromatin and its availability to the action of therapeutic agents, thereby contributing to the development of the mechanism of chemoresistance [490].

6-phosphofructo-2-kinase is an enzyme that catalyzes another irreversible stage of glycolysis: the conversion of fructose-6-phosphate to fructose-1,6-bisphosphate [491]. As with most other glycolytic enzymes, the activity and expression of 6-phosphofructo-2-kinase are impaired in various cancers and are strongly correlated with aggressiveness and poor prognoses. It was found that the PFKFB3 gene, which encodes the third isoform of 6-phosphofructo-2-kinase and is a key effector protein of transforming growth factor β1 (TGFβ1), stimulates glycolysis in the pancreatic carcinoma cell line Panc1, thereby mediating the epithelial–mesenchymal transition necessary for the acquisition of the invasive ability of cells [492]. A similar situation, along with strengthening of the epithelial–mesenchymal transition mediated by the activation of NF-κB pathway signaling, is shown in gastric carcinoma, where high levels of expression of the PFKFB3 gene are positively correlated with tumor size, differentiation, invasion, metastasis, and poor patient survival [493]. In the work conducted by Moon et al., it was shown that the constitutive activation of 6-phosphofructo-2-kinase, which is found in the cells of L-lymphatic nodular carcinoma of the prostate, leads to a high rate of glycolysis and, as a result, plays an important role in stimulating androgen-induced lipogenesis [494], a process necessary for the proliferation of neoplastic cells [495]. The role of 6-phosphofructo-2-kinase as an activator of the glycolytic rearrangement of neoplastic cells is also shown in cancers of the central nervous system. Thus, the levels of the PFKFB3 gene are significantly higher in high-grade gliomas (G3 and G4 degrees of differentiation) than in non-pathological brain tissues or gliomas with G1 and G2 degrees of differentiation [496].

Pyruvate kinase is an enzyme that catalyzes the third physiologically irreversible glycolysis reaction, namely, the conversion of phosphoenolpyruvate to form one molecule of pyruvate and generate one ATP molecule. Among the various isoforms, the M2 isoform (PKM2) has attracted the most attention as a target for potential anti-neoplastic therapeutic agents due to its increased expression in transformed cells [497]. Since pyruvate kinase M2 is widely expressed during embryogenesis and regeneration, it is clear that its enzymatic activity abilities are extremely important in actively proliferating cells, such as tumor cells [498]. As a prognostic and diagnostic marker of malignant neoplasms, PKM2 plays a critical role in maintaining the metabolic program of neoplastic cells in non-muscle-invasive and highly differentiated muscle-invasive bladder carcinomas [499], colorectal cancer [500], ovarian carcinoma [501], etc. In addition to its involvement in the development of solid tumors, in an evaluation of the blood plasma of patients with acute myeloid leukemia, acute lymphoblastic leukemia (ALL) showed significantly higher levels of PKM 2, the values of which were negatively correlated with the prognosis of survival [502]. Moreover, Wang et al. found that the oncogenic functions of PKM2 are implemented not only because of the ability of the enzyme to enhance glycolysis but also to mediate the activation of autophagy by increasing the phosphorylation of the key protein of the early process-initiation Beclin-1 [503], contributing to cell survival in AML.

Since the exposure to the glycolysis process in general and directly to glycolytic enzymes can increase the efficacy of existing treatment strategies by sensitizing tumor cells to therapy, the selective targeting of specific enzymes or isoforms of enzymes is a promising area for the development of new anticancer drugs.

### 4.3. Correction of Anomalies in Oxidative Phosphorylation in Mitochondria as a Promising Therapeutic Approach in the Development of Neuroprotective Drugs

Maintaining mitochondrial integrity and function is of the highest priority for neuronal cells, as neurons are both key consumers of ATP and provide its highest yield [504]. There is strong evidence demonstrating that, as a result of oxidative phosphorylation disorders, the cerebral hypometabolism precedes clinical manifestations of neurodegenerative conditions [505]. This provides a rationale for corrections aimed at the metabolism and mitochondrial function constituting a potential strategy for modifying the diseases by blocking their progression.

The analysis of brain specimens from patients with various forms of affective and neurodegenerative pathologies shows disorders of mitochondrial functions as dysfunctions of electron transport and oxidative phosphorylation. In particular, the NADH dehydrogenase and cytochrome C oxidase complexes are particularly impaired in bipolar disorder [506,507], schizophrenia [508,509], Parkinson’s disease [510,511], and Alzheimer’s disease [512,513]. A large-scale study by Lunnon et al. [514] showed that a significant decrease in the activity of complex I was observed in temporal cortex specimens obtained from patients with Alzheimer’s disease; this was accompanied by the lower expression of the NDUFA1/4/7-9, NDUFB2/3/6, and NDUFS3/4/5 subunits in the blood. Meanwhile, the activity of cytochrome C oxidase was lower in the frontal, motor, occipital, parietal, and temporal lobes of the cerebral cortex, as well as in the hippocampal regions, compared to clinically healthy controls. It is clear that these lobes of the brain are potentially involved in cognitive decline in Alzheimer’s disease [515,516,517,518], suggesting their critical contribution to neurodegeneration. Perluigi et al. [519] reported a close correlation between the level of mitochondrial dysfunction and neuronal death in the hippocampal and polysensory regions of the neocortex, as well as in the entorhinal cortex.

The role of the other three complexes—succinate dehydrogenase, cytochrome reductase, and ATP synthase—in the formation of the pathological phenotype of these diseases should not be underestimated, but, to date, they have been studied to a much lesser extent than complexes I and IV. Nevertheless, interesting results were obtained in parallel evaluations of the activity of NADH + cytochrome with reductase complexes in the work of Francis et al. [520], a study conducted in transgenic TgCRND8 mice at 45 weeks of age. The authors were able to identify bioenergetic insufficiency in the brains of animals modeling Alzheimer’s disease, comprising a decrease in the activity of complexes I and III and an impairment of ATP levels. A proteomic study of specimens of frozen brain tissue from patients with this neurodegenerative disorder showed a pattern of low activity of complexes I and III and ATP synthase in the early stages of Alzheimer’s disease, while dysfunction of the cytochrome C oxidase complex was observed in later stages [521]. In turn, a study by Emmerzaal et al. [522] showed an age-related decrease in mitochondrial complex II activity in APPswe/PS1ΔE9 mice aged 9 to 14 months. Interestingly, the cognitive dysfunction observed in animals of this line was observed exactly in the age interval of 8–10 months [523], which suggests the critical role of this mitochondrial abnormality in cognitive decline in Alzheimer’s disease. Another study showed a parallel decrease in the activity of the succinate dehydrogenase complex in the brains of APP/PS1 mice [524], confirming the significant contribution of this complex to the energy function of mitochondria.

There are numerous disputes about whether anomalies in the functioning of the mitochondrial electron transport chain occur at the earliest stages of disease development, even before the appearance of any histopathological abnormalities, or whether they depend on the direct influence of previous pathological signs, mainly toxic deposits of β-amyloid peptide (Aβ). The way in which the interaction between mitochondria and Aβ influences the pathogenesis of Alzheimer’s disease is still largely unknown. There is a significant amount of evidence that Aβ, accumulating in the mitochondrial matrix, plays an important role in mitochondrial collapse and the neuronal damage mediated by the dysfunction of these organelles in Alzheimer’s disease [525,526,527,528]. By inhibiting the enzymatic activity of complexes III and IV, toxic forms of β-amyloid peptide significantly inhibit mitochondrial respiration, thereby reducing the production of ATP [529]. In addition, Aβ has been shown to be colocalized with the α subunit of ATP synthase in the cortex and hippocampal regions of the brains of mice modeling Alzheimer’s disease, leading to a decrease in extracellular ATP levels due to damage to the electron transport chain and, as a result, increased long-term depression in synapses [530]. Meanwhile, the opposite situation has been well proven, when mitochondrial dysfunction provokes Aβ toxicity [531,532]. In particular, in the studies of Emmerzaal [522] and Djordjevic [533], decreases in the activity of complex II in APP/PS1 animal models and protein levels in all five subunits of electron transport chain complexes in 3xTg mice were observed even before the appearance of β-amyloid plaques, detected later in life. In addition, the critical role of impaired NADH dehydrogenase activity in the pathological Aβ mechanisms has been proven in a cell model of SH-SY5Y that overexpresses the Swedish mutation of the human amyloid precursor protein, where rotenone-induced dysfunction of mitochondrial complex I increased β-amyloid toxicity [534], allowing us to consider mitochondrial dysfunction as the starting point of the amyloid cascade.

The literature is also rich in examples showing the synergy of another biomarker of Alzheimer’s disease, phosphorylated tau protein, and defects in electron transport chain complexes in the formation of disease pathogenesis [535]. In an in vivo model of genetically modified animals overexpressing the mutant human tau protein P301L, the accumulation of hyperphosphorylated tau led to a decrease in the activity of the NADH–dehydrogenase complex and impaired ATP production [536]. Later, Rhein et al. demonstrated the tau-dependent dysregulation of complex I in transgenic pR5 mice at the age of eight months [537] with abnormally phosphorylated protein [538], thus confirming the close relationship between the two main pathological features of Alzheimer’s disease. In turn, Yamada et al. [539] showed that the injection of the mitochondrial complex I inhibitor annonacin in mice expressing the R406W-tau mutation led to an increase in the number of neurons with hyperphosphorylated tau protein in the frontal and parietal lobes, as well as in the hippocampal region of the brain.

However, regardless of which process has the function of a trigger, nowadays, there are extremely powerful arguments proving that an impairment of oxidative phosphorylation causes a general bioenergetic crisis in neurons, leading to cell death under neurodegenerative conditions. Moreover, since the normal function of neuronal cells requires well-coordinated mechanisms, which, in turn, require an appropriate supply of energy substrates, focusing on the pathological process as a therapeutic target constitutes a sensible direction in the development of potential neuroprotective drugs.

### 4.4. Determination of the Main Metabolic Processes of the Cell and Energy Metabolism

Targeting the glycolysis process is an appealing prospect for therapeutic interventions in the treatment of malignant neoplasms. To date, several approaches to modulating metabolism in tumor cells have been outlined (Table 3).

One of the most obvious strategies is the use of agents that block the glucose transporter GLUT, thereby preventing glucose from entering the cell and disrupting the glycolytic pathway.

Just over 10 years ago, Liu et al. identified a small molecule inhibitor, GLUT1 WZB 117 [540], of which studies of the antitumor activity continue to this day. During this period, research teams were able to detect a wide range of antineoplastic properties in WZB117 in various models of malignant neoplasms. In particular, in their first work [540], the authors reported that, by blocking GLUT1, this compound deprives transformed cells of glucose as an energy source and reduces the levels of intracellular ATP and glycolytic enzymes, which are accompanied by cell cycle arrest and the necrotic death of neoplastic cells. All of this mediated a reduction in tumor size in NU/J nude mice with a xenograft model of non-small-cell lung cancer by more than 70%. Similar properties of WZB117 were shown in an evaluation of the effect of the compound on the regulation of the metabolism and the viability of the neuroblastoma cell line [541]. Thus, due to the effect on GLUT1, the treatment of SH-SY5Y with WZB117 reduced the contents of glycolytic metabolites and ATP, inducing cell cycle arrest and the initiation of cell death up to necrosis. Notably, as a specific inhibitor of GLUT1, WZB117 also inhibits the self-renewal of cancer cells [542], one of the typical properties of which is intense glycolysis [552].

Years later, the range of positive properties of WZB117 was expanded, as evidenced by a number of studies on its chemosensitizing properties [544,553,554,555,556], as the involvement of GLUT1 in the development of drug resistance is well known [557,558]. In particular, the use of WZB117 allowed colon cancer cells to overcome resistance to the action of 5-fluorouracil [555], Adriamycin [556], and radiation [544] in the case of the human breast cancer cells MCF-7/ADR and MDA-MB-231/MCF-7, imatinib in gastrointestinal stromal tumors [559], and apatinib against the human melanoma cells A375 and SK-MEL-28 [543].

Ginsenoside GRg 3, derived from *Panax ginseng*, has the ability to significantly reduce the proteins GLUT 1 (~50%) and GLUT4 (~40%), which are overexpressed in tumor cells HGC-27 (derived from the metastatic lymph nodes of gastric carcinoma) and AGS (atypical glandular cells of gastric cancer) [560]. Clearly, this may be one of the possible mechanisms discovered earlier for GRg3 antitumor properties. Interestingly, in a recently published study by Zhu et al. [545], GRg 3 was used as an active ingredient to develop unique liposomes loaded with the known cytostatic paclitaxel (Rg3-PTX-LP). Thus, by targeting GRg3 to GLUT1 [561], the authors were able to reverse the resistance of cells of tumorous origin MCF 7/T to paclitaxel and achieve more than 90% tumor inhibition.

In the work of Chen et al. [562], owing to comprehensive in vitro and in vivo analyses of the antitumor properties of SMI 277—a new modulator of GLUT1—the authors were able to detect the high inhibitory ability of the compound in relation to the process of glucose uptake, which was confirmed by a decrease in lactate production. This mechanism of action of SMI277 effectively inhibited proliferation and triggered the apoptotic death of the transformed cells, which, in in vivo experiments in a mouse model, was accompanied by a decrease in tumor size by more than 50%.

Based on the above findings, it is easy to see that studies of the antitumor properties of the current GLUT inhibitors are in the initial stages. To date, the FDA has not approved a single GLUT modulator, and its development and implementation in clinical practice remain serious problems due to the widespread expression of these proteins and the impossibility of targeting only those localized in tumor cells. This has led to the use of alternative approaches in modulating the metabolism of malignant neoplasms; among these, the effect on the key glycolytic enzymes mentioned in the previous subsection is promising.

As one of the promising inhibitors of hexokinase 2, lonidamine was recently assessed, passing the third phase of clinical trials, but it was never introduced into clinical practice due to its high systemic toxicity [563]. However, the literature is rich in experimental data obtained in the study of the antitumor properties of lonidamine derivatives. The introduction of the lonidamine motif to the structure of organometallic ruthenium led to a significant increase in cytotoxic properties in relation to the MCF 7 cell line, mediated by the activation of apoptotic caspases [564]. Additionally, the combination of cisplatin, tegafur, and lonidamine in one molecule demonstrated excellent cytotoxic properties on the triple-negative breast cancer cell line due to DNA damage and the disruption of the metabolic state of cells [565].

The role of jasmonates in the inhibition of hexokinase 2 and, as a result, their exertion of antitumor effects, have been well described in the literature [566]. In particular, the derivative methyl jasmonate represents a promising strategy for the treatment of multiple myeloma [546]. Thus, treatment with a compound of cells obtained from patients with this disease led to the impaired activity of HK2, a decrease in ATP production, and subsequent death due to the dysfunction of intracellular oncogenic signaling pathways. These properties of methyl jasmonate have prompted researchers to consider the possibility of using it as an adjuvant therapy. In the work of Klippel et al. [546], the combination of methyl jasmonate with bortezomib, which is a known proteasome inhibitor [567], demonstrated synergistic action in cell models of multiple myeloma MM.1S and INA6, which may indicate the possibility of using this agent in the treatment of hematological diseases. The chemosensitizing properties of methyl jasmonate have also been shown when combined with 5-fluorouracil, Adriamycin, and sorafenib in the treatment of hepatocellular carcinoma [547]. Of particular interest are the results obtained in [546], where the authors found that the inhibitory effect of methyl jasmonate on the glycolysis process is selective for cells of tumorous origin, which explains the absence of severe damage to the liver, lungs, and kidneys, and is an absolute advantage for future clinical use.

There is a relatively small pool of data on the inhibitors of two other key glycolysis enzymes, 6-phosphofructo-2-kinase and pyruvate kinase. However, in the work of Jiang et al. [548], (2 E)-3-(3-Pyridinyl)-1-(4-pyridinyl)-2-propen-1-one (3 PO), as a modulator of PFKFB3 activity, led to a decrease in lactate production and in the expression of the anti-apoptotic proteins survivin, c-IAP1, and c- IAP 2, as well as the activation of the NF-κB-mediated signaling pathway, leading to the death of A2780CP ovarian cancer cells. The chemotherapeutic potential of 3PO was also confirmed in [568], where the inhibition of PFKFB3 resulted in the effective suppression of the proliferation of a number of cell lines of tumorous origin, which was accompanied by a decrease in the intracellular concentrations of fructose-2,6-bisphosphate, lactate, and ATP. Despite the positive properties of 3PO described above, the mechanism of PFKFB3’s inhibitory action is questionable, as recent studies have found that this agent does not interact with PFKFB3, and the effects it shows may not be due to the action of this enzyme at all [569,570]. This set the direction for new work in the field, prompting researchers to design selective modulators of 6-phosphofructo-2-kinase; AZ67 [571] was developed as a result, the uniqueness of which is explained by the fact that, due to its targeted binding to PFKFB3, AZ67 in critically low doses implements an antiangiogenic effect without affecting glycolysis.

Approximately 100 publications were found in the PubMed search engine when processing the query “shikonin-pkm2”. Thus, it is possible to comprehensively analyze the promising results obtained by teams of authors on the inhibition of pyruvate kinase by this representative of the M2 isoform and its associated therapeutic effects. Among the most prominent is the work of Wang et al. [549], in which, due to the addition of shikonin to the standard treatment regimen for bladder cancer, the overactivation of PKM 2 was convincingly proven. The authors were able to enhance the therapeutic effect of cisplatin in cytostatic-resistant T24 cells. This ability of shikonin to lead to cell death due to necrosis or apoptosis was accompanied by disturbances in the regulation of proteins of the Bcl-2 family. A similar effect has also been shown in a model of non-small-cell lung cancer, where the increased sensitivity to cisplatin by shikonin may be due to the inhibition of exosome secretion [550]—direct participants in the formation of the resistant phenotype of tumor cells [572]. The inhibition of pyruvate kinase activity by shikonin in an ovarian cancer model enhanced the antitumor properties of Olaparib [501], reaffirming the adjuvant potential of a naturally occurring product. In turn, the evaluation of the effects of shikonin in esophageal squamous cell carcinoma in an in vivo xenotransplant model showed the ability of the agent to inhibit cell proliferation by blocking PKM 2 and, as a result, aerobic glycolysis by regulating the PKM 2/STAT3 signaling pathway [551]. A pool of data was discovered for shikonin, describing the current state of research on its antitumor properties due to its PKM2-inhibitory ability; these data allow us to anticipate the rapid introduction of the agent into preclinical and clinical trials in order to confirm its pharmacological prospects and bring to the market a new drug with an exceptional therapeutic profile.

As mentioned earlier, disorders of energy metabolism are a typical feature of a wide range of diseases, especially neurodegenerative disorders. The central target for the action of therapeutic agents with a neuroprotective type of activity is mitochondria, the dysfunction of which is involved in various pathological cascades in NDD. Meanwhile, at first glance, the concept of strengthening mitochondrial bioenergy by activating the work of electron transport chain complexes seems obvious. In particular, for the metabolite of soy isoflavone daidzein S-equol, the ability to potentiate the functions of brain mitochondria was found to arise due to the positive regulation of the activity of the IV complex [573], which directly correlates with the protective effect of the compound in the modeling of Aβ-induced neurotoxicity [574].

However, an equally interesting approach in the development of neuroprotective drugs is the production of drugs with an inverse function, the so-called “soft” inhibitors of mitochondrial respiration. The positive effects exhibited by such agents may be due to two phenomena: (1) the decrease in the production of mitochondrial ROS and (2) the development of adaptive mechanisms acquired by organelles in response to a kind of positive mobilizing stress. One of the most prominent examples of such compounds is a drug with antihyperglycemic activity: metformin. As a reversible inhibitor of the activity of the NADH dehydrogenase complex, metformin leads to the induction of chaperone-mediated autophagy in transgenic APP/PS1 mice, which mediates a decrease in the number of Aβ deposits in the brain and prevents the development of a pathological behavioral phenotype [575]. Moreover, a number of clinical trials have already been conducted that aim to evaluate the effect of metformin on the cognitive functions of patients. In particular, in a meta-analysis performed by Zhang et al. [576], it was found that this drug significantly improved the neurobehavioral profile of patients with type 2 diabetes.

Thus, based on the above findings, it can be concluded that a number of studies have indeed demonstrated the efficacy of the therapeutic approach that consists of modulating metabolic shifts in malignant neoplasms and neurodegenerative disorders, thereby confirming its scientific justification. However, the preclinical and clinical evaluations of metabolic modulators for the treatment of these diseases remain in a relatively embryonic condition, which only increases the interest in continuing research in order to explore the potential of therapeutic options. Already, we can be sure that the development of highly specific drug agents, aimed at the metabolism of transformed cells in cancer diseases and neurons in neurodegenerative disorders, will create a completely new range of tools for use against these diseases.

## 5. Conclusions

The relationship between oncological diseases and neurodegenerative disorders is extremely complex. However, in recent years, convincing scientific data have been accumulated indicating the contribution of a number of etiological factors and pathophysiological processes to the pathogenesis of these two radically different diseases, thereby demonstrating an intriguing relationship between oncology and neurodegeneration.

Based on our detailed review of critical molecular mechanisms that simultaneously contribute to the development of both types of diseases, several important points can be identified.

The conceptual origin of the term “oxidative stress” can be traced back to the second half of the twentieth century. It was then that the researchers wondered about the fleeting nature of free radicals and the molecular mess caused by their action. In this regard, for more than 50 years, the concept of oxidative stress has largely focused only on the biology of free radicals, considered trigger factors of a cascade of pathological reactions associated mainly with the aging process. In recent years, the idea of oxidative stress has evolved significantly. It has been proven that the increase in its intensity leads to fatal consequences in a wide range of chronic and acute diseases, especially, paradoxically, two diametrically different pathologies: oncological and neurodegenerative. Moreover, over the past 20 years, the relevant research has focused on the most promising paradigms based on the components of the cell’s own intrinsic antioxidant defense system and intracellular signaling pathways regulating the production of free radicals. Because free radicals are key regulatory factors in the molecular mechanisms of the pathogenesis of malignant neoplasms and neurodegenerative disorders, targeting various chains of oxidative stress can help to determine a rational therapeutic strategy for these diseases. The development of effective therapeutic strategies aimed at modulating oxidative stress in various types of diseases is an important link in the creation of treatment protocols. The most promising profile of this activity type has been demonstrated for products of natural origin. Due to direct or indirect regulation of intracellular levels of reactive oxygen species, they are able to have therapeutic effects. In this regard, the modification of compounds of this class, which are valuable sources of biologically active molecules, is certainly considered as a priority direction in the creation of drugs.

Epigenetic modifications are another molecular determinant that has obvious cross-functional pathways in the pathogenesis of oncological and neurodegenerative diseases. Remarkably, the curiosity shown in the middle of the last century about the enzymatic activity that catalyzes the removal of acetyl groups from histone proteins of calf thymus extract turned into the scientific community’s pronounced interest in the field of epigenetics, playing a critical role in understanding the pathogenetic mechanisms of various diseases. As a result, there is already convincing evidence that a number of expression patterns of various HDAC isoforms are altered in people with oncological diseases; this plays a direct role in the manifestation of numerous pathological changes. As a result of such epigenetic modifications, neoplastic cells manage to maintain a highly proliferating tumor phenotype. At the same time, there is unequivocal evidence that HDACs regulate signaling cascades, gene activity, and the expression levels of other molecules involved in the pathogenesis of neurodegenerative diseases. It also demonstrates positive epidemiological correlations between oncology and neurodegeneration. Because there is a clear relationship between HDACs and the pathological phenotype in these human diseases, therapeutic agents that are effective against these enzymes are considered to be innovative treatment options both for monotherapy and in combination with already known drugs for the treatment of oncological and neurodegenerative disorders. Current HDAC inhibitors have rather low specificity, which limits their therapeutic effectiveness due to significant side effects. In this regard, one of the priority directions in the creation of HDACi as therapeutic agents for neurodegenerative and oncological diseases treatment is the development of selective agents targeting specific isoforms of the enzyme. This feature could lead to the active targeting of specific pathological signs and increase the chances of candidates for success in the clinic.

Disorders in cellular metabolism are also of fundamental importance in future research in the search for additional biomarkers and the development of new therapeutic strategies for the treatment of these diseases. Approximately 30 Nobel Prizes have been awarded for work related to the studies of the mechanisms of metabolic reactions, the understanding of which has, over hundreds of years (since the first mention in the work of Ibn al-Nafis—XIII century AD), expanded from individual enzymes and metabolites to complex pathways that have a close relationship and dependence on each other. Such discoveries allowed us to define metabolism in detail, which helped us to understand metabolic changes in various human diseases. To date, the range of pathological conditions associated with disorders in the glycolysis and oxidative phosphorylation processes has been described in many review and experimental papers. Thus, the bioenergetic hypothesis of the pathogenesis of malignant neoplasms covers a variety of altered metabolic pathways that provide tumor cells with the energy necessary for intensive proliferation and resistance to apoptosis. This is achieved due to the property of metabolic heterogeneity, when neoplastic cells, producing ATP, are equally oriented to aerobic glycolysis and oxidative phosphorylation. In turn, in neurodegenerative diseases, mitochondrial respiration is a vulnerability, the dysfunction of which describes successive events leading to a decrease in cognitive function ranging from metabolic deficiency to neuronal death. In this regard, the multifaceted modulation of metabolic pathways is considered a modern therapeutic solution for the treatment of these diseases. A number of experimental and preclinical studies have convincingly proven the role of cellular metabolism modulators in the treatment of cancer and neurodegenerative disorders. Based on our analysis of existing data, a critical conclusion can be drawn: real clinical success of such drugs can only be achieved by developing agents with a very high degree of molecular target specificity; which is mainly due to the ubiquity of metabolic enzymes and transporters.

Thus, increasing the awareness of researchers based on our comprehensive analysis of the possible relationship between oncological diseases and neurodegenerative disorders, as well as a review of the current trends in research and in the clinical application of the most promising therapeutic platforms of various chemical structures, can contribute to the development of both advanced diagnostic tools and innovative methods of treating these diseases.

## Figures and Tables

**Table 1:** Therapeutic potential of natural products as modulators of oxidative stress in the treatment of oncological and neurodegenerative diseases.

Therapeutic Agent	Molecular Mechanisms of Action	Prognostic Significance	Disease	
Quercetin	Inhibition of the PI3K/Akt signaling pathway	Restoration of sensitivity of transformed cells to docetaxel action [197]	PC	Oncological diseases
	Reduction in the expression of oxidative stress markers and restoration of the leukocyte count	Reduction in inflammation, reduction in tumor size [206]	CC	
	Regulation of the PI3K/Akt/mTOR signaling pathway and inhibition of Nrf2 expression	Reversing drug resistance to cisplatin [202]	EOC	
Quercetin + Vitamin C	Decreased Nrf2 expression and activity of glutathione enzymes	Stimulation of tumor cell death [203,204]	BC	
Artesunate	ROS-dependent cell cycle arrest due to changes in cyclin D3, E2F-1, and p21 expression	Antiproliferative effect [223]	EOC	
	Induction of ROS-dependent apoptosis by reducing the VDAC and increasing the cleavage of caspase 3		NSCLC	
	Cell cycle arrest due to an increase in p16, p21, p-IRE1a, and LC3B and a decrease in Ki67 and cyclin D1		CC	
	Inhibition of NF-kB, ubiquitin-mediated degradation of castration-resistant prostate cancer cells	Reversal of cell resistance to androgen receptor antagonists [226]	PC	
Costunolid	Triggering of apoptosis as a result of ROS hyperproduction and φ_m_ violation	Transformed cell death [232,233,234]	BCa	
			ESCC	
			BC	
Parthenolide	PI3K/Akt pathway blocking, ROS hyperproduction	Antiproliferative action [235,236,237]	CCa	
	GSH depletion, NF-kB shutdown		BC	
	Increase in ROS production, decreased GSH activity		LN	
Quercetin	Inhibition of LPO, increase in GSH expression	Danio rerio cognitive dysfunction restoration in PTZ-induced neurodegeneration [208]	AD	Neurodegenerative diseases
	Reducing the COX-2 level	Improvement of memory parameters in mice with LPS-induced neurodegeneration [209]		
	Activation of Nrf2, decrease in the MDA level, increased expression of SOD, CAT, and GSH	Preventing neuronal damage, leveling cognitive impairment in rats with Alzheimer’s disease model stimulated by toxic Aβ_1-42_ forms [210]		
Artemisinin	Decrease in the MDA level, increased SOD and GSH expression	SH-SY5Y cell death inhibition in MPP^+^-induced neurotoxicity [227], reduction in damage to dopaminergic neurons with MPTP-induced toxicity [228]	PD	
	Blocking of ROS production as a result of Akt signaling pathway activation	Increased HT-22 survival with glutamate-induced neurotoxicity [229]	AD	
	Activating the ERK/CREB pathway	Inhibition of SH-SY5Y cell death in Aβ_1-42_ toxicity, 3xTg transgenic mice cognitive function improvement [231]		
Artemeter	Activation of the Nrf2 signaling pathway, decrease in the level of inflammatory mediators, Aβ levels, and activity of β-secretase 1	Inhibition of neuroinflammation in LPS-stimulated BV2 microglia [230]		
Costunolid	Decrease in the intracellular ROS level and caspase 3 expression	Preventing damage to the PC12 cell line by H_2_O_2_ [238]		
Parthenolide	Blocking of the AKT/MAPK/NF-kB signaling pathway, neuroinflammation reduction	Improvement of memory indicators in the APP/PS1 transgenic mice line [239]		
	Inhibition of MAO B activity	Cell death decrease in MPP^+^-induced toxicity [240]	PD	

Abbreviations: PC, pancreatic cancer; CC, colorectal carcinoma; EOC, epithelial ovarian carcinoma; BC, breast cancer; NSCLC, non-small-cell lung cancer; PC, prostate cancer; BCa, bladder cancer; ESCC, esophageal squamous cell carcinoma; CCa, cervical cancer; LN, lymphoid neoplasms; AD, Alzheimer’s disease; PD, Parkinson’s disease; PTZ, pentylentetrazole; LPS, lipopolysaccharide; VDAC, voltage-dependent anion-selective channel 1; SOD, superoxide dismutase; CAT, catalase; GSH, reduced form of glutathione; MAO B, monoamine oxidase B; MPP^+^, 1-methyl-4-phenylpyridinium; MPTP, 1-methyl-4-phenyl-1,2,3,6-tetrahydropyridine.

**Table 2:** Therapeutic potential of vorinostat as an adjuvant agent in the treatment of oncological and neurodegenerative diseases.

Therapeutic Agent in Combination	Molecular Mechanisms of Action	Prognostic Significance	Disease	
^131^I-methaiodbenzylguanidine	Increase in human NET protein expression	Increased radioligand absorption and frequency of true response [412,427,428]	NB	Oncological diseases
Isotretinoin	Modulation of APF2 levels	Five-year progression-free and overall survival improvement [413,429,430]	MB	
Hydroxychloroquine	Autophagy inhibition due to increased cathepsin D and p62 levels	Strengthening of antitumor immunity [414,415,431]	CC	
Pazopanib	Degradation of mutant p53, increased VEGF expression, decreased HIF-1α levels	Increase in the average duration of overall survival and life without progression of the disease [416,432]	EOC	
			BC	
			CC	
			GC	
			HNSCC	
			NSCLC	
Chemoradiotherapy	Increased apoptosis rate due to increased Bax and p21 expression	Improved overall survival [417,418,419]	HNSCC	
			PC	
			GB	
Rosiglitazone	Increased expression of neurotrophic factor genes	Reduced biochemical, cellular, and behavioral disorders in the STZ mouse model of Alzheimer’s disease [424]	AD	Neurodegenerative diseases
Rapamycin	Decreased APP due to increased expression of Beclin 1, neurotrophic factors GDNF, BDNF, NGF, and neuronal markers MAP2 and LAMP2	Relief of cognitive dysfunction in rats with an insulin resistance and intracerebroventricular injection Aβ_1-42_ [425]		
Tadalafil	Restoration of long-term potentiation, Aβ, and tau pathology relief through the Akt/GSK3β pathway	Restoration of cognitive functions in APP/PS1 transgenic mice [426]		

Abbreviations: NB, neuroblastoma; MB, medulloblastoma; CC, colorectal carcinoma; EOC, epithelial ovarian carcinoma; BC, breast cancer; GC, gastric carcinoma; NSCLC, non-small-cell lung cancer; HNSCC, head and neck squamous cell carcinoma; PC, pancreatic cancer; GB, glioblastoma; AD, Alzheimer’s disease; NET, norepinephrine transporter; APF2, angiotensin converting enzyme; VEGF, vascular endothelial growth factor; HIF-1α, hypoxia-induced factor 1-α; APP, amyloid precursor protein; GDNF, glial neurotrophic factor; BDNF, brain neurotrophic factor; NGF, nerve growth factor; MAP2, microtubule-associated protein 2; LAMP2, lysosome-associated membrane protein 2; A β_1-42_, pathological form of β-amyloid peptide 1-42.

**Table 3:** Therapeutic potential of promising modulators of glycolytic function.

Therapeutic Agent	Key Target	Molecular Mechanisms of Action	Prognostic Significance	Disease	
WZB117	GLUT1	Cell cycle arrest, necrotic cell death	Tumor size reduction in a xenograft mouse model [540]	NSCLC	Oncological diseases
			Decreased cell viability [541]	NB	
		Self-renewal of stem cell obstruction	Tumor initiation inhibition in a xenograft mouse model [542]	PC	
		Blocking the STAT3/PKM2 pathway	Overcoming resistance to apatinib [543]	M	
		AMPK activation, blocking the mTOR pathway, increased Bax and Bcl-2 translocation in mitochondria	Increased sensitivity to Adriamycin and radiation [544]	BC	
		Decreased AKT and Bcl-2 expression	Overcoming resistance to imatinib [543]	GIST	
GRg3	GLUT1, GLUT4	IL-6/STAT3/p-STAT3 pathway inhibition, MDSC suppression, CAF and collagen fibers decrease, cell death	Overcoming resistance to paclitaxel in an in vivo xenograft model [545]	BC	
Methyl jasmonate	HK2	Decrease in the level of AKR1C1	Induction of cell death, overcoming resistance to bortezomib [546]	MM	
		Opening of the mPTP due to dissociation of the HK2/VDAC1 complex, triggering apoptotic cell death	Increased cell sensitivity to 5-fluorouracil, Adriamycin, and sorafenib in a xenograft mouse model [547]	HCC	
3PO	PFKFB3	Decreased survivin expression, c-IAP1 and c-IAP2, NF-κB activation	Cell death induction [548]	EOC	
Shikonin	PKM2	Decrease in Bcl-2 expression, apoptotic cell death	Increasing the therapeutic effect of cisplatin [549,550]	BC	
		Exosome secretion inhibition		NSCLC	
		DNA damage, decreased in BRCA1	Overcoming resistance to Olaparib [501]	EOC	
		PKM2/STAT3 pathway inhibition	Reduced tumor growth in an in vivo xenograft model [551]	ESCC	

Abbreviations: NSCLC, non-small-cell lung cancer; NB, neuroblastoma; PC, pancreatic cancer; M, melanoma; BC, breast cancer; GIST, gastrointestinal stromal tumor; MM, multiple myeloma; HCC, hepatocellular carcinoma; EOC, epithelial ovarian carcinoma; BC, bladder cancer; ESCC, esophageal squamous cell carcinoma; AMPK, 5′AMP-activated protein kinase; MDSCs, myeloid-derived suppressor cells; CAF, cancer-associated fibroblasts.

## Data Availability

Not applicable.
